# Seasonal Variation in the Faecal Microbiota of Mature Adult Horses Maintained on Pasture in New Zealand

**DOI:** 10.3390/ani11082300

**Published:** 2021-08-04

**Authors:** Karlette A. Fernandes, Erica K. Gee, Chris W. Rogers, Sandra Kittelmann, Patrick J. Biggs, Emma N. Bermingham, Charlotte F. Bolwell, David G. Thomas

**Affiliations:** 1 School of Agriculture and Environment, College of Sciences, Massey University, Private Bag 11-222, Palmerston North 4442, New Zealand; Karlette@wvs.org.uk (K.A.F.); c.w.rogers@massey.ac.nz (C.W.R.); 2 School of Veterinary Science, College of Sciences, Massey University, Private Bag 11-222, Palmerston North 4442, New Zealand; e.k.gee@massey.ac.nz (E.K.G.); p.biggs@massey.ac.nz (P.J.B.); c.bolwell@massey.ac.nz (C.F.B.); 3 AgResearch Ltd., Grasslands Research Centre, Palmerston North 4442, New Zealand; sandra.kittelmann@sg.wilmar-intl.com (S.K.); emma.bermingham@agresearch.co.nz (E.N.B.)

**Keywords:** bacteria, community ecology, diversity, faeces, pasture, horse, Illumina MiSeq, microbiota, next-generation sequencing, nutrient composition, ribosomal RNA gene, water-soluble carbohydrates

## Abstract

**Simple Summary:**

Ten horses were kept on pasture for one year, with hay provided from June to October. Each month we measured how much pasture was present and collected pasture and hay samples to assess their nutrient content, and faecal samples from all horses to investigate the diversity of the bacterial species present using next-generation sequencing technology. The population of faecal bacteria was more diverse during the months when the horses were kept exclusively on pasture compared to the months when pasture was supplemented with hay. The diet offered, and the season and the month we sampled the paddock all had a major influence on the diversity of the species of bacteria in the faeces. While there were some differences between the horses, generally the bacterial populations could be grouped together in samples obtained during May, June, and July (late-autumn to winter period), and January, February, and March (a period of drought). More specifically we were able to show an association between specific bacterial species, nutrients (dry matter, protein, and structural carbohydrates), and climatic conditions (rainfall and temperature). Our study showed that the diversity and composition of the bacterial population of horses kept on pasture changes over a 12-month period, and this reflects changes in the nutrient composition of the pasture, which in turn is influenced by climate. The findings of this study may have implications for managing horses on pasture and the use of forages for horses susceptible to digestive problems.

**Abstract:**

Seasonal variation in the faecal microbiota of forage-fed horses was investigated over a 12-month period to determine whether the bacterial diversity fluctuated over time. Horses (*n* = 10) were maintained on pasture for one year, with hay supplemented from June to October. At monthly intervals, data were recorded on pasture availability and climate (collected continuously and averaged on monthly basis), pasture and hay samples were collected for nutrient analysis, and faecal samples were collected from all horses to investigate the diversity of faecal microbiota using next-generation sequencing on the Illumina MiSeq platform. The alpha diversity of bacterial genera was high in all samples (*n* = 118), with significantly higher Simpson’s (*p <* 0.001) and Shannon-Wiener (*p <* 0.001) diversity indices observed during the months when horses were kept exclusively on pasture compared to the months when pasture was supplemented with hay. There were significant effects of diet, season, and month (ANOSIM, *p <* 0.01 for each comparison) on the beta diversity of bacterial genera identified in the faeces. While there was some inter-horse variation, hierarchical clustering of beta diversity indices showed separate clades originating for samples obtained during May, June, and July (late-autumn to winter period), and January, February, and March (a period of drought), with a strong association between bacterial taxa and specific nutrients (dry matter, protein, and structural carbohydrates) and climate variables (rainfall and temperature). Our study supports the hypothesis that the diversity and community structure of the faecal microbiota of horses kept on pasture varied over a 12-month period, and this variation reflects changes in the nutrient composition of the pasture, which in turn is influenced by climatic conditions. The findings of this study may have implications for grazing management and the preparation of conserved forages for those horses susceptible to perturbations of the hindgut microbiota.

## 1. Introduction

Horses thrive on high-forage diets due to the microbial fermentation of fibre and other compounds in the hindgut [1]. This digestive strategy requires a high food intake level (above 30 g kg^−1^ body weight^0.75^ day^−1^ [2]) to maintain proper gastrointestinal function, with forage comprising the major component of the diet. Below this threshold level of forage intake, nutrient supply to the hindgut microbiota becomes the major constraint of digestive function and efficiency [2]. Research has shown that the balance of microbial populations in the hindgut is important to maintain digestive health, immune function, and performance of the animal [3,4,5]. Over the past decade, research has focused on understanding the structure and composition of these microbial communities in the equine hindgut (using caecal and faecal samples) [4,6]. Several studies have indicated that the structure of the equine hindgut microbiota is complex and is comprised of a highly diverse community dominated by bacteria, amongst other microbial species such as archaea, protozoa and fungi [6,7,8,9].

Some studies have indicated that changes observed in the relative abundance of faecal bacteria may be associated with dietary modifications and gastrointestinal disturbances [10,11,12], which theoretically also occur prior to the onset of life-threatening conditions like colic and laminitis [13,14,15]. Colic and laminitis are two of the most common problems that affect horses [16], so the potential to predict the onset of sub-clinical gastrointestinal disturbances by examining changes in the abundance of faecal bacteria has been suggested as a method that could perhaps prevent the sudden onset of clinical signs of disease [13].

A previous study conducted by our group [7] reported that the faecal bacterial community of forage-fed horses was highly diverse, and the profile was diet-specific with significant differences observed in the relative abundance of certain bacterial genera (the most dominant one in both diets was an unclassified genus within the family Ruminococcaceae). Furthermore, when horses housed indoors and maintained on a commercial chopped ensiled forage were moved outside to graze on pasture, alterations in the bacterial community profile were observed within four days. Although the gut microbiota of the horses appeared to adapt quickly to the new (pasture) diet, some fluctuation (both an increase and/or decrease) in the beta diversity of bacterial communities were observed over the 3-week study period, which was hypothesised to be due to changes in pasture composition [7].

While many horses around the world are stabled and fed formulated compound diets, the major feed source for New Zealand horses is pasture, mostly consumed directly from the paddock, but also after conservation as ensiled forages and hay [17,18,19]. A temperate climate enables continuous growth of pasture in New Zealand, and many horses graze on pasture all year round [17,18,19,20]. While there is vegetative grass-leaf throughout the year, the proportion of dead-leaf increases during late-summer (February) and autumn (March–April), and during plant stress due to drought or freezing temperatures [21,22]. Thus, there are seasonal changes in the dry matter (DM) and macronutrient composition of the pasture. While one study used conventional microscopic enumeration to compare the faecal microbiota of horses kept on summer versus winter pastures in Japan [23], there have been very few studies that have used metagenomic techniques to examine the dynamics of bacterial communities in the faeces of horses maintained on pasture [24].

Given the expected seasonal fluctuations in pasture composition, we hypothesised that there is an effect of time (month or season) on the profile of the faecal bacterial community of pasture-fed horses. The aims of the current study were to investigate changes in the structure and composition of faecal bacterial communities over a 12-month period, by collecting cross-sectional snapshot data at monthly intervals and to correlate the changes in microbiota populations with the macronutrient composition of pasture over the study period.

## 2. Materials and Methods

### 2.1. Ethics Statement

The animals used in the study were part of Massey University’s teaching herd. All faecal samples were collected during the routine per-rectal examinations organised to teach students enrolled in the undergraduate veterinary degree program. The use of animals, including the welfare, husbandry, and handling complied with the code of ethical conduct for the use of live animals for research, testing, and teaching (Massey University Animal Ethics Committee, Palmerston North, New Zealand; Teaching Protocol number 11/100).

### 2.2. Experimental Design

#### Animal Details and Management

Ten mares (eight Standardbred and two Thoroughbred, median age 15 years, interquartile range (IQR) 12–19 years), managed as a cohort at the Veterinary Large Animal Teaching Unit, Massey University, were enrolled in the study from April 2013 to March 2014. At the beginning of the trial, the mares had a mean body weight of 494 ± 44 kg, a median body condition score (BCS) of 5 (IQR 5–6) measured on a 9-point scale [25], and a median cresty neck score (CNS) of 2 (IQR 1–3) measured on a 6-point scale [26]. The mares were barren (non-pregnant) and were not “in work” during the study period. The horses were set stocked in a 2.5-ha paddock that contained a standard New Zealand pasture mix of predominantly ryegrass and white clover species (~80–95% perennial ryegrass (*Lolium perenne*) and 5–20% white clover (*Trifolium repens*)) [16]. The average sward height of the pasture was 4 ± 1 cm at the start of the study. The horses received unrestricted access to pasture (ad libitum) throughout the year and were kept in three similar two-hectare paddocks rotated every 5–6 months. Due to seasonal reduction in pasture growth from June to October, the diet was supplemented with hay (ryegrass-clover hay mix), which was offered to the horses in the paddock twice daily at the rate of 6–10 kg DM/horse/day. The hay was harvested from a single location (Manawatu, New Zealand) in January 2013, processed as one batch, and stored in a barn for use during the study. The horses had ad libitum access to water in self-filled troughs in the paddock. Faecal egg counts were performed on all horses every 4–6 weeks, followed by administration of individual dose-dependent anthelmintic treatments if necessary (Equitak Paste, Bayer Animal Health, Auckland, New Zealand).

### 2.3. Sample Collection

#### 2.3.1. Faecal Sample Collection

A faecal sample was collected from each horse at monthly intervals between 1000 and 1200 h on a single day. The faecal material was collected per-rectum, a sub-sample was transferred into a 2 mL polyethylene cryogenic vial (Ray Lab Ltd., Auckland, New Zealand), and snap-frozen in liquid nitrogen immediately. The samples were transferred to a −80 °C freezer within two hours of collection and stored until laboratory analysis. A total of 120 faecal samples (10 horses × 12 months) were collected during the study period.

#### 2.3.2. Feed Sample Collection

Representative pasture samples were collected from the paddock each month around midday, on the same days as the faecal samples were collected. According to previously described pasture sampling techniques [21], the grazing behaviour of the horses was observed to identify sites used to estimate the grazing height of the pasture (height of stubble remaining after grazing). Within the paddock, fifty pasture-sampling sites were selected by walking in a zigzag manner through the paddock and selecting a site every 20 steps. At each site, approximately 20–50 g of pasture was cut (at the pre-estimated grazing height of ~1 cm above the ground) and collected in a polythene bag that was placed in an icebox. After collecting approximately 1 kg of pasture, the sample was mixed thoroughly and transferred to a zip-lock plastic bag. During the months from June–October, representative hay samples (500 g) were collected by taking multiple grab-samples from each hay bale (core and outer surface), which were also mixed thoroughly before transfer to zip-lock plastic bags. All samples were weighed (recorded as fresh weight) and stored at −20 °C until further processing within 2–4 h of collection. At six-monthly intervals, the samples were lyophilised (FD18, Cuddon Freeze Dry, Blenheim, New Zealand) and ground to pass through a 1 mm screen (Cyclotec 1093 Sample Mill, Foss, Hillerod, Denmark) [27,28]. The ground samples were stored at −20 °C until laboratory analysis to evaluate the nutrient content of the feed samples, which was performed at the end of the study period.

#### 2.3.3. Laboratory Analysis DNA Extraction, Amplicon Library Construction, and Sequencing

Nucleic acids were extracted from 100 mg of each faecal sample (*n* = 120) using a combined bead-beating, phenol-chloroform, and column purification protocol and QIAquick 96 PCR purification kit (Qiagen, Hilden, Germany) [29,30], with some modifications (Supplementary Materials, Text S1). The extracted and purified gDNA was eluted in 80 μL elution buffer (10 mM Tris; pH 8.5 with HCl). All gDNA samples were quantified using the NanoDrop ND-1000 UV-Vis Spectrophotometer (NanoDrop Technologies, Wilmington, DE, USA) and the quality was assessed by visualisation of high molecular weight bands on a 1% (*w*/*v*) agarose gel. A subset of samples (*n* = 12) was quantified with Quant-iT dsDNA HS, RNA, and Protein assay kits (Invitrogen, Carlsbad, CA, USA) on a Qubit 2.0 Fluorometer (Invitrogen, Carlsbad, CA, USA) to assess the quality of gDNA and to check for the presence of RNA and protein contamination.

The samples were normalised to 5 ng/μL gDNA per sample and bacterial 16S rRNA gene libraries were constructed using the Illumina two-step PCR library preparation method (Text S1; New Zealand Genomics Limited, Massey Genome Service, Palmerston North, New Zealand). Briefly, for each gDNA sample, the V3–V4 hypervariable region of the 16S rRNA gene was targeted using the universal primer pair S-D-Bact-0341-b-S-17; S-D-Bact-0785-a-A-21 [31], because these sub-regions are the most reliable regions for representing the full-length 16S rRNA sequences, with a superior phylogenetic resolution of most bacterial phyla [32,33]. The amplicons were attached to the Illumina adapter overhang nucleotide sequences (forward and reverse) in the amplicon-PCR step. This was followed by ligation with a unique 8 bp dual-index barcode sequence (Nextera XT DNA library preparation kit, Illumina, San Diego, CA, USA) in the index-PCR step, for individual sample identification (Table S1). Amplification was performed on a Thermocycler ProS (Eppendorf, Hamburg, Germany), with an initial denaturation at 95 °C for 3 min, 25 cycles of denaturing (95 °C for 30 s), annealing (55 °C for 30 s) and elongation (72 °C for 30 s), and a final 5-min extension at 72 °C. At each PCR step amplicons were generated using a KAPA HiFi PCR kit (KapaBiosystems, Wilmington, MA, USA), purified using a magnetic bead capture kit (AMPure, Agencourt, Beckman Coulter, Beverly, MA, USA), and quantified on a Qubit 2.0 Fluorometer (Invitrogen, Carlsbad, CA, USA), to check the quality of gDNA (Quant-iT dsDNA HS assay kit, Invitrogen, Waltham, MA USA) and the presence of contamination (Quant-iT RNA and Protein assay kits, Invitrogen, Waltham, MA USA). Following validation of the purified sequence libraries using a DNA 1000 labchip on a 2100 Bioanalyzer (Agilent Technologies, Santa Clara, CA, USA), the 16S metagenomic sequence libraries (*n* = 120) were pooled in equimolar concentrations into two pools with 60 libraries each. The pooled libraries were denatured using fresh NaOH, and spiked with 10% volume of a PhiX control library (PhiX control kit v3, Illumina, San Diego, CA, USA), before loading onto 2 × 250 base paired-end sequencing runs (60 libraries per run, samples from January–June on run 1 and July to December on run 2) using the Illumina MiSeq platform (MiSeq 500 cycle kit, v2 chemistry, Illumina, San Diego, CA, USA) [34,35,36].

#### 2.3.4. Analysis of Nutrient Composition

The pasture (*n* = 12) and hay (*n* = 5) samples were processed using analytical chemistry methodologies (Nutrition Laboratory, Massey University, Palmerston North, New Zealand) for the quantitative determination of Dry Matter (DM) (AOAC 930.15; convection oven 105 °C, Contherm 2000, Contherm Scientific Ltd., Lower Hutt, New Zealand), and Ash (AOAC 942.05; Furnace 550 °C, Elecfurn:Muf 25/20/40, The Electric Furnace Co Ltd., Auckland, New Zealand). Total Nitrogen (N) was determined by the Dumas method [37] and was converted to Crude Protein (CP) by multiplying by 6.25 (AOAC 968.06; CNS 2000, LECO Corporation, St. Joseph, MI, USA). Fat was determined by the Soxhlet extraction method (AOAC 991.36; Tecator Soctec System HT, 1043, FOSS, Hillerod, Denmark). Fibre analysis including Neutral Detergent Fibre (NDF), Acid Detergent Fibre (ADF), and Lignin was determined using the ANKOM TDF Fiber Analyser (ANKOM Technology, Macedon, NY, USA), and Hot Water Soluble Carbohydrate (HWSC) content was determined by the Nelson-Somogyi assay [38,39]. The Gross Energy (GE) content was determined through the heat of combustion (Gallenkamp Adiabatic Bomb Calorimeter, Loughborough, Leicestershire, UK). Other feed values were calculated as follows: Total carbohydrates (CHO) = 100 − (CP + fat + ash); non-structural carbohydrates (NSC) = 100 − (CP + fat + ash + NDF); and Digestible energy (DE MJ/kg DM) = 2118 + 12.18 (CP) − 9.37 (ADF) − 3.83 (NDF−ADF) + 47.18 (fat) + 20.35 (NSC) − 26.3 (Ash) × 0.00418 [40,41].

### 2.4. Data Recording and Analysis

The general health, feeding, and management of the horses were monitored daily by the farm manager. During the monthly collection of faecal samples for microbiota analysis, a subjective assessment of body condition and cresty neck scores was made by one of the authors (KAF). The pasture cover was also monitored on a monthly basis by measuring the average sward height (cm ± SD) of the pasture by using a standard metric ruler in the areas being grazed by the horses at the time of faecal and feed sample collection, The recorded sward height was compared with a custom scale (Farmax sward stick, Hamilton, New Zealand) to estimate the pasture herbage mass (kg DM/ha) available to the horses. Daily recordings of temperature and rainfall were retrieved from the National Climate Database (National Institute of Water and Atmospheric Research-NIWA, Auckland, New Zealand), which contained data from a weather station situated ~2 km from the paddock location (Palmerston North EWS, coordinates 40.38195° S 175.60915° E, AgResearch Grasslands Ltd.). The data for temperature and rainfall were averaged to present monthly recordings.

#### 2.4.1. Bioinformatics Analysis

Quality control analysis was performed on the original sequences using three processes: SolexaQA++ (http://solexaqa.sourceforge.net/, accessed on 1 February 2015), FastQC (http://www.bioinformatics.babraham.ac.uk/projects/fastqc/, accessed on 1 February 2015) and FastQscreen (http://www.bioinformatics.babraham.ac.uk/projects/fastq_screen/, accessed on 1 February 2015). The raw sequence reads obtained from the Illumina MiSeq runs were aligned against the PhiX genome using BWA (http://bio-bwa.sourceforge.net/, accessed on 1 February 2015), and the PhiX sequences detected were removed, leaving the unaligned sequences that were included in further downstream analysis. The SAM (Sequence Alignment/Map) files generated from BWA and the fastq files were reconstructed using SAM Tools (available at http://broadinstitute.github.io/picard/, accessed on 1 February 2015). Illumina adaptors and PCR primers were removed using the FASTQ processing utilities “fastq-mcf” program (available in the ea-utils suite of tools version 1.1.2-621, https://code.google.com/p/ea-utils/, accessed on 1 February 2015). The sequence reads were assigned to corresponding samples by examining the 8 bp barcode sequence [36], and the read pairs were extracted and concatenated according to the barcodes for each paired read from each sample, by joining together the overlapping reads using the FLASh software (version 1.2.11, http://ccb.jhu.edu/software/FLASH/, accessed on 1 February 2015), which generated the best-aligned contigs [42]. The processed sequences were trimmed to their longest contiguous segment for which error probabilities were greater than a threshold of 0.003 (equivalent to the quality of ~25 Phred score) using the DynamicTrim application from the SolexaQA++ software (version 3.1.2, http://solexaqa.sourceforge.net/, accessed on 1 February 2015) and short reads (<250 bp) were removed from the bacterial sequence library using the LengthShort application [43]. All sequences that did not meet the above quality filtering criteria were excluded from further downstream analysis. The project is registered with NCBI PRJNA286058, and the sequence data generated in this study are available via the Sequence Read Archive under the accession number SRA272143.

Ecological analysis on the retained sequence data was performed using the QIIME package (Quantitative Insights Into Microbial Ecology, v1.8) [44]. Clustering of operational taxonomic units (OTUs) was performed using the uclust method at a 97% similarity threshold and potential chimeras were removed using the usearch61 option in the QIIME scripts (*pick_otus.py*–m usearch61) [45]. Representative OTU sequences were aligned using PyNAST [46] and assigned to taxonomic ranks [47] by a BLAST-search against the Greengenes core reference alignment database for bacterial 16S rRNA genes [48].

To access the richness of bacterial taxa captured within the samples, Collector’s curves were constructed from the original OTU tables generated in QIIME, using the *alpha_diversity.py* script and the “observed species” metric. The alpha diversity rarefaction analysis was computed for a maximum of 19,250 sequences per sample and was visualised for each parameter included in the metadata (Table S1). Subsequently, the original OTU table was rarefied to a subsample of 19,250 sequences per sample, in an attempt to decrease bias caused by non-uniform sequencing depth [49]. Good’s coverage (mean percentage ± SD) was estimated to ensure representative subsampling [50], which was calculated in QIIME using the *alpha_diversity.py* script with the “goods coverage” metric and summarised using MS Excel (version 2010, Microsoft Corp., Redmond, WA, USA).

Alpha diversity was evaluated at the genus level using the PAST software (version 3.08, http://folk.uio.no/ohammer/past/, accessed on 1 February 2015) [51], and included the Simpson’s index of diversity [52], Shannon-Wiener index of entropy [53] and the Chao1 index for the richness of bacterial genera [54]. Relative abundances of bacterial taxa were summarised at phylum and genus levels. Bacterial phyla and genera with relative abundances <1% in all samples were grouped as “other phyla” and “other genera”, respectively. The taxonomic profiles for the bacterial phyla and genera were visualised on heatmaps using MetaPhlAn (version 1.7.8, http://huttenhower.sph.harvard.edu/metaphlan, accessed on 1 February 2015) [55]. Beta diversity was evaluated on a genus level using the QIIME pipeline [44] and included only those bacterial taxa that represented ≥1% of the total community, in at least one sample. Differences between bacterial communities were determined using Bray-Curtis dissimilarity, which takes into account the presence or absence of a species and the relative abundance. Principal coordinate analysis (PCoA) was performed in QIIME and the clustering of samples based on the first three principal coordinates was visualised using EMPeror [56], SigmaPlot (version 13, Systat Software, Inc., San Jose, CA, USA) and MEGAN5 MEtaGenome ANalyzer (version 5, http://ab.inf.uni-tuebingen.de/software/megan5/, accessed on 1 February 2015), as required. Unweighted Pair Group Method with Arithmetic Mean (UPGMA) clustering was performed in QIIME, based on the Bray-Curtis dissimilarity index, to visualise the hierarchical clustering of samples by season and month. The dendrograms were visualised using MEGA6 (version 6.0) [57] and MEGAN5 software tools, (version 5.0).

#### 2.4.2. Statistical Analyses

The data generated in QIIME and PAST were imported into MS Excel and re-formatted where necessary before tests for statistical significance were conducted. Statistical analysis was performed using the SAS v9.4 (SAS Institute Inc., Cary, NC, USA), STATA v12.1 (Stata Corp., College Station, TX, USA), and R (www.r-project.org, accessed on 1 February 2015) software packages. All variables were checked for normal distribution using the Shapiro-Wilk test (significance value of *p <* 0.05). All data are presented as mean ± SD for parametric data or median ± IQR for non-parametric data. Seasons were classified as follows: Autumn—March, April, May; Winter—June, July, August; Spring—September, October, November; and Summer—December, January, February.

Differences for the alpha diversity indices between groups (diet, horse, month, and season), and for the median Bray-Curtis dissimilarity indices between the within- and between-horse variation in each diet group and between-horse variation within each diet, were determined using the Kruskal-Wallis test with a Steel-Dwass correction for multiple sample comparisons (as required) at a significance value set at *p <* 0.05 (proc npar1way Wilcoxon dscf in SAS). The effect of diet, horse, season and month on the beta diversity of bacterial taxa were tested using the Analysis of Similarity (ANOSIM) option with the QIIME script *compare_categories.py*. Significant differences between the relative abundance of bacterial taxa were determined by the *group_significance.py* script in QIIME using the Kruskal-Wallis test with Bonferroni adjustment for multiple comparisons (adjusted *p* values were *p <* 0.003 for phylum level and *p <* 0.001 at genus level comparisons).

Correlations between the four most dominant bacterial taxa with nutrient and climate variables were determined by Pearson’s Correlation with P values that were calculated and plotted in R using the packages hmisc (http://cran.r-project.org/web/packages/Hmisc, accessed on 1 February 2015) and corrplot (http://cran.r-project.org/web/packages/corrplot, accessed on 1 February 2015). For the correlation analysis of nutrient variables versus microbiota abundance, data on the nutrient composition of pasture from only those months when pasture was the sole component of the diet were included. The four dominant bacterial genera selected were renamed as follows: Genus1 (unclassified genus within family Ruminococcaceae), Genus2 (unclassified genus within order Bacteroidales), Genus3 (unclassified genus within order Clostridiales), and Genus4 (unclassified genus within family Lachnospiraceae).

## 3. Results

### 3.1. Health and Condition of the Horses

The median BCS of the horses during the study was 5 (IQR 4–6) (*p =* 0.96) and the median CNS was 2 (IQR 1–3) (*p =* 0.99), with no significant differences observed during the 12-month study period. The horses remained clinically normal throughout the study period. There were no reports of gastrointestinal or musculoskeletal issues during the experimental timeframe and none of the horses received antibiotic treatment during the study period.

### 3.2. Forage and Climate Monitoring

The macronutrient composition of the pasture and hay samples recorded at monthly intervals during the 12-month study period is shown in Table 1. The nutrient composition of pasture varied during the study period. The %DM in the pasture was the highest in February (late-summer) and March (early-autumn), which coincided with the lowest rainfall and highest temperatures recorded during the drought period. Correspondingly high values for %CHO, %NDF and %ADF content and low values for %CP and %HWSC content were also observed in the pasture during this drought period (January–March). With rising temperatures and rainfall in spring, the sward height and pasture cover increased, the %HWSC increased to the highest values in October–November, and %NSC values peaked in late spring (October–November) and early summer (December). An increase in NSC was also observed in early autumn (March) following the drought period in summer (January–February) (Table 1).

The %DM in the hay was relatively consistent (ranging from ~95 to 96% DM), but there was some variation in the %CP (ranging from ~9–15%) observed across the five sampling time-points in the same batch of hay (Table 1). There was also variation in the %HWSC (~6–9%), %NDF (~43–63%), %ADF (~30–37%), and %NSC (~16–30%). However, the variation observed in the hay samples was lower than the variation observed in the pasture samples (Table 1).

### 3.3. Comparison of the Faecal Microbiota (Bacterial Communities)

#### 3.3.1. Metrics of Sequencing Data and Rarefaction Analysis

The two runs on the Illunima MiSeq platform generated ~16 million sequences. On preliminary examination of the data, one sample (collected from Horse 6 during the month of August) had a significantly lower number of sequences (*n* = 2808 reads) than other samples (*n* ≥ 19,250 reads). On preliminary phylogenetic analysis, another sample (collected from Horse 9 during the month of May) had a significantly higher proportion (96.9%) of proteobacteria when compared to the other samples (0.1–1.6%). These two samples were outliers, and hence, were removed from downstream phylogenetic analysis (dataset of *n* = 118 faecal samples). The mean number of sequences per sample and the metrics of data for sequences that passed quality filtering are shown in Table 2. After normalisation at 19,250 sequences per sample, the total number of OTUs detected at 97% similarity across all samples was 85,725. The mean number of OTUs per sample was 4819 (range 3521–6499). A total of 2,271,500 bacterial sequences obtained from the samples (*n* = 118) were included in the downstream analysis, wherein at least 33 bacterial phyla were detected, which encompassed at least 90 different taxonomic classes, 170 orders, 323 families, and 646 genera.

**Figure 1:**
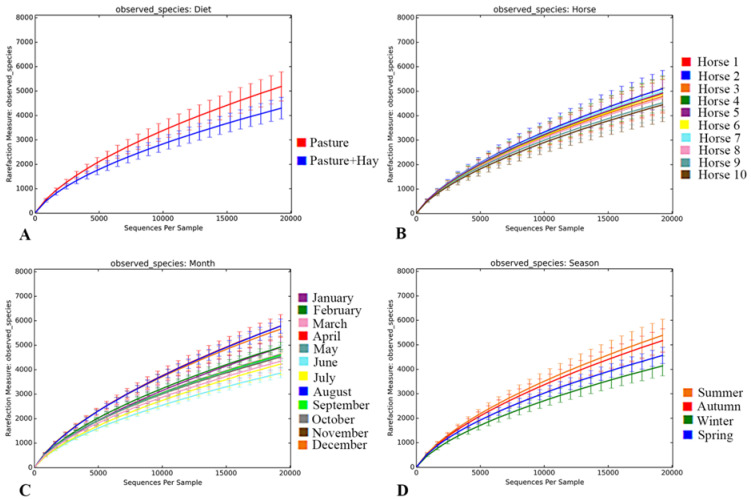
Alpha rarefaction curves illustrated by diet, horse, month, and season. Each rarefaction curve was generated using the observed species metric for up to 19,250 sequences per sample. The panels represent the rarefaction curves for diet (**A**), horse (**B**), month (**C**) and season (**D**). The diet periods were categorised as “Pasture” when the horses were grazed exclusively on pasture, and “Pasture + Hay” when the horses were grazed on pasture and supplemented with hay in the paddock. Seasons were categorised as follows: Summer—December, January, February; Autumn—March, April, May; Winter—June, July, August; Spring—September, October, November.

The rarefaction curves generated using the observed species metric for alpha diversity of the bacterial sequences are displayed in Figure 1. New OTUs were identified at the end of sub-sampling at 19,250 sequences per sample, which may indicate a lack of complete sampling effort. However, the rate of new OTU discovery was relatively limited around that sub-sampling threshold. Good’s coverage estimates on the normalised OTU table indicated that the sampling depth had adequately captured a large part of the OTU diversity in all samples, with the mean coverage being 84 ± 3%. Figure 1 also indicated that there were some differences in the diversity within the faecal bacterial community for diet, season, and month.

#### 3.3.2. Diet-Specific Differences in Diversity of the Faecal Bacterial Community

The Pasture and Pasture + Hay diets showed a high diversity of bacterial genera detected in the faeces. The Simpson’s and Shannon-Wiener diversity indices were significantly higher (*p <* 0.001) during the months when horses were kept exclusively on pasture versus pasture supplemented with hay. This was reflected in the trend for differences in the richness of bacterial genera (Chao1) between the two time periods (Table 3).

**Figure 2:**
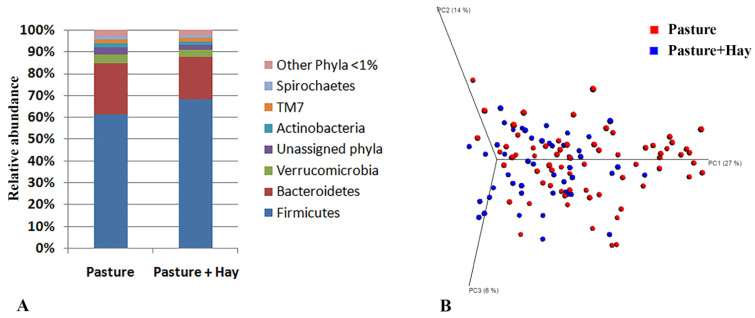
Comparison of the faecal bacterial community across diet periods. (**A**) Mean relative abundance of bacterial phyla identified in the faecal samples of horses fed Pasture versus Pasture + Hay. (**B**) 3-D graph of principal coordinate analysis (PCoA) showing beta diversity of the bacterial community in the faeces of horses fed Pasture and Pasture + Hay. The first three PCs explained 47% of the variation (27% by PC1, 14% by PC2 and 6% by PC3).

The bacterial community of both diets comprised 33 phyla, 14 of which were present at ≥1% abundance, and the remaining 19 phyla (other phyla) had abundances <1% (overall 15 taxa for comparisons at the phylum level). The bacterial community was dominated by two phyla, the Firmicutes (Pasture 62% and Pasture + Hay 68%) and the Bacteroidetes (Pasture 23% and Pasture + Hay 19%), which together accounted for >80% of the overall abundance of bacterial phyla (Figure 2, panel A). A total of 646 bacterial genera were identified, 51 of which had abundances ≥1% and the remaining 595 genera (other genera) had abundances <1% (overall 52 taxa for comparisons at genus level). The most abundant bacterial genera in both of the diet periods were an unclassified genus within the family Ruminococcaceae (20–23%), an unclassified genus within the family Lachnospiraceae (13–14%), an unclassified genus within the order Clostridiales (12–14%) and an unclassified genus within the order Bacteroidales (9–11%), which accounted for ~54–62% of the overall abundance of bacterial genera.

There were significant effects of diet periods on the beta diversity of bacterial genera (ANOSIM, *p* = 0.002, R^2^ = 0.122), which appeared to cluster by diet period on the principal coordinate analysis, with 47% of the variation explained on three principal coordinates (27%, 14% and 6% on PCs 1, 2 and 3, respectively) (Figure 2, panel B). This clustering by diet period was further described by significant differences observed in the relative abundances of seven bacterial phyla (Table S2) and 12 bacterial genera (Table S3).

#### 3.3.3. Inter- and Intra-Horse Variation

There was a significant effect of the horse on the beta diversity of bacterial genera (ANOSIM, *p =* 0.002, R^2^ = 0.067). The median Bray-Curtis dissimilarity index for the period when pasture was fed (0.21 (IQR 0.17–0.26)) was greater than the Pasture + Hay diet period (0.20 (IQR 0.17–0.24)) (*p =* 0.001). This pattern was seen for between-horse (*p =* 0.006) and within-horse (*p =* 0.024), comparisons. Differences in the beta diversity observed between horses were reflected in the significant differences in the relative abundances of several bacterial taxa. At the phylum level, the relative abundances of only three less abundant phyla were significantly different between the horses (Table S4). The relative abundances of the most dominant phyla (Firmicutes and Bacteroidetes) did not differ. At the genus level, the relative abundances of the two genera differed between the horses, and these did not include the most dominant genera (Table S5).

**Figure 3:**
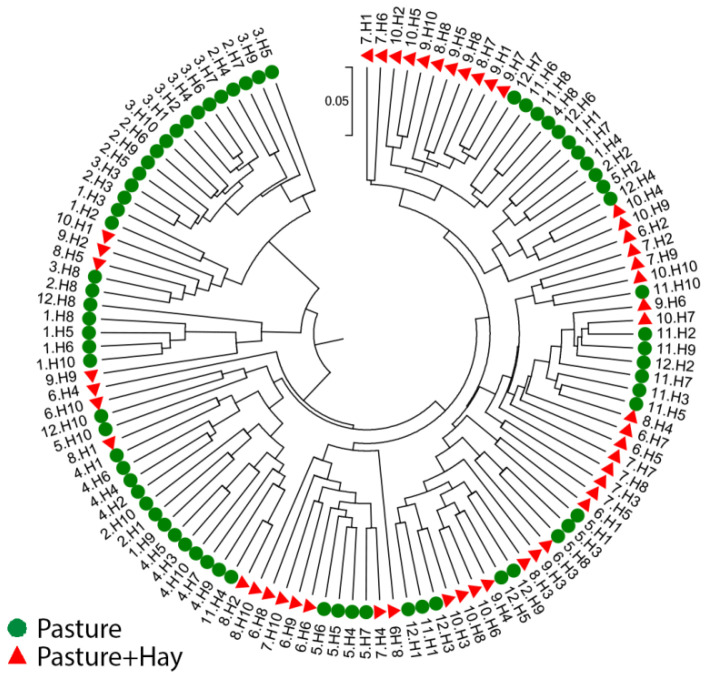
The bacterial community structure of faecal samples (*n* = 118) included in the study. The circular cladogram showing beta diversity of the faecal sampleswas constructed using Unweighted Pair Group Method with Arithmetic Mean (UPGMA). The sample labels are coloured by diet periods and consist of a month number (1–12 representing January–December) and horse number (H1–H10).

Within each diet period, there was a significant difference in the median Bray-Curtis dissimilarity index between individual horses (Pasture *p <* 0.007 and Pasture + Hay *p <* 0.001), indicating the presence of some variation in the bacterial communities between individual horses (Table 4). However, the median between-horse dissimilarity was similar to the median within-horse dissimilarity (Pasture *p =* 0.758 and Pasture + Hay *p =* 0.115), indicating that the significant effect of the horse on the beta diversity of bacterial genera may be due to temporal factors. The UPGMA cladogram constructed using all samples included in the study shows some clustering of horses by diet periods and indicates the possibility of effects due to temporal factors (Figure 3).

#### 3.3.4. Temporal Effects on the Diversity of the Faecal Bacterial Community

All seasons and months showed high values for the alpha diversity of bacterial genera. There was a significant difference in the Simpson’s diversity index between seasons, with significantly higher median diversities observed in autumn compared to summer, winter, and spring (Table 5). The median Shannon-Wiener indices were significantly higher in summer and autumn than in winter and spring, whereas the richness of bacterial genera (Chao1 index) was similar in summer, autumn, and winter, but significantly lower in spring. Significant differences in alpha diversity indices were also observed between the 12 months of the study period (Table 6).

**Figure 4:**
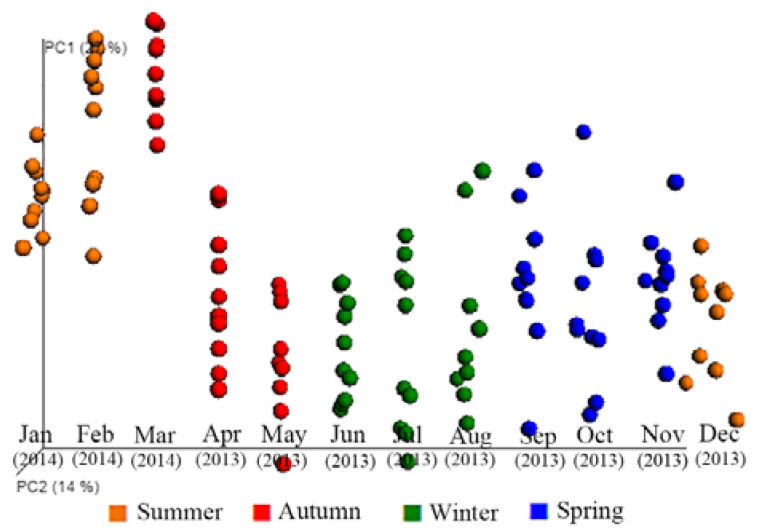
PCoA of the beta diversity of faecal bacteria in horses illustrated by season over a 12-month period. The graph shows the principal coordinate analysis (PCoA) of the beta diversity in the bacterial community; with each faecal sample represented as a dot (i.e., each dot represents a horse within a month). The principal coordinates explain 27% (PC1) and 14% (PC2) of the variation. The months of the year are shown on the horizontal axis and seasons are colour coded and categorised as follows: Summer—December, January, February; Autumn—March, April, May; Winter—June, July, August; Spring—September, October, November.

**Figure 5:**
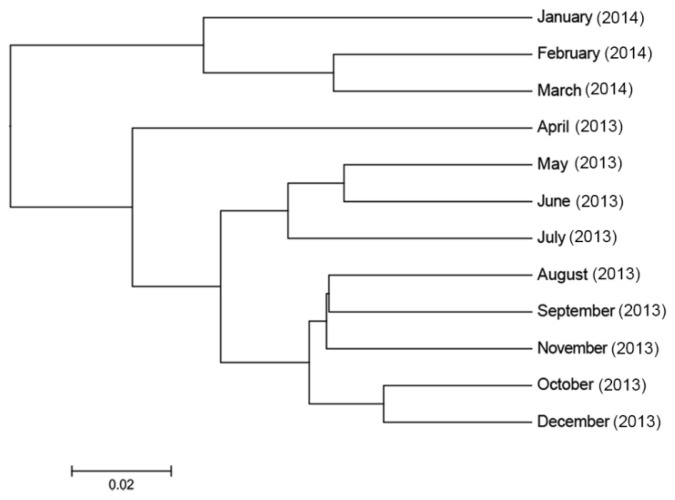
Month-wise hierarchical clustering of the faecal bacterial community of horses included in the study. The cladogram shows the beta diversity of the bacterial community in the faecal samples and was constructed using Unweighted Pair Group Method with Arithmetic Mean (UPGMA).

There were significant effects of the season (ANOSIM, *p =* 0.001, R^2^ = 0.190) and month (ANOSIM, *p =* 0.001, R^2^ = 0.479) on the beta diversity of bacterial genera in the faeces. Although there appears some overlap on the PCoA graphs, clustering by season (Figure S1, panel A) and month (Figure S2, panel B) was observed, where most (~47%) of the variation was explained on three principal coordinate axes. Figure 4 illustrates the beta diversity of the bacterial genera detected in the faeces of the 10 horses, grouped by season. The dispersion of the individual samples on PC1 indicates that there was some variation in the beta diversity among the 10 horses. However, shifts in the beta diversity were evident across the seasons, with one cluster observed in the last three months of the study during the drought period (January, February, March), a second cluster during the late autumn and winter months (May, June, July) and a third cluster comprising the remaining months of the year (Figure 4). The hierarchical clustering of the bacterial communities observed on PCoA was confirmed by the separation of clades observed for the three clusters on the UPGMA cladogram constructed for the samples grouped by month (Figure 5). The bacterial community structure in autumn and summer (dryer pasture) originated from a different clade to that of spring and winter (greener pasture) (Figure S2).

**Figure 6:**
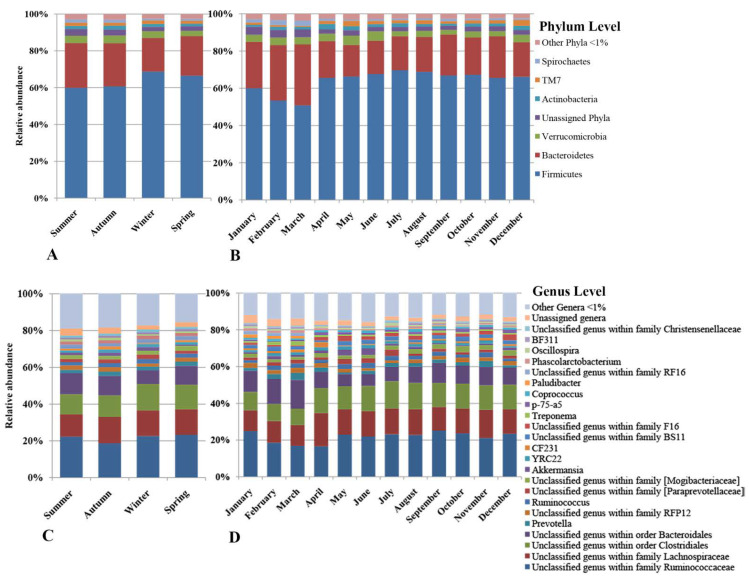
Comparison of relative abundances of the faecal bacteria of horses included in the study. The relative abundances of the bacterial community are presented at phylum and genus levels. The stacked bar charts in panels (**A**,**C**) show the relative abundances by seasons, and panels (**B**,**D**) show the relative abundances by month. Phyla and genera with mean relative abundances of <1% were categorised as other phyla <1% or other genera <1%.

The temporal effects and hierarchical clustering (by season and month) observed in the bacterial community structure were also described by differences in the relative abundances of several bacterial taxa at the phylum and genus levels (Figure 6). Across seasons, five bacterial phyla and 21 genera were significantly different (Tables S6 and S7, respectively), while across months, 11 bacterial phyla and 39 genera were significantly different (Tables S8 and S9, respectively). In both the season and month comparisons, significant differences were observed between most of the dominant bacterial phyla and genera.

#### 3.3.5. Correlation between Nutrient Composition, Climate Variables and Relative Abundance of Faecal Bacterial Genera

**Figure 7:**
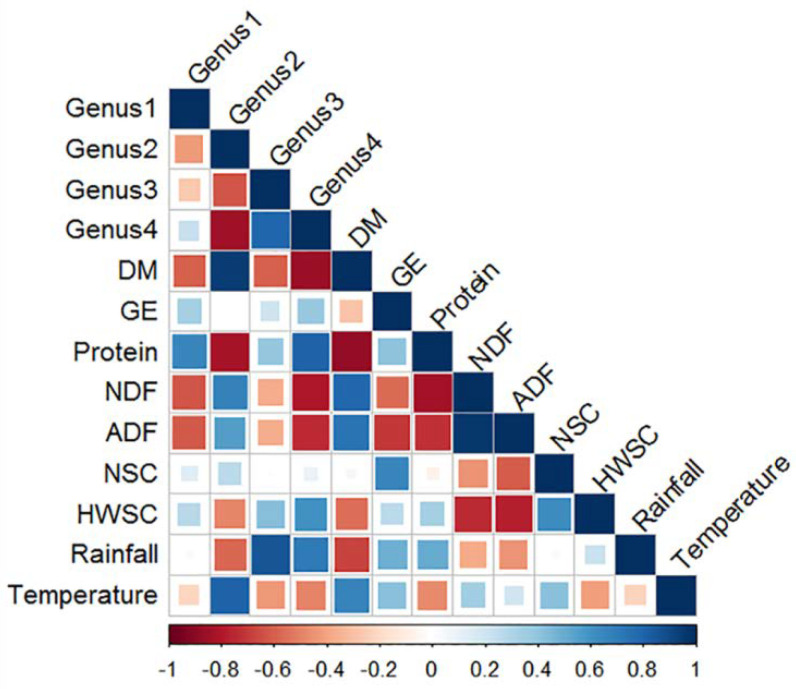
Correlation matrix of bacterial taxa, macro nutrients, and climate variables. Each square on the matrix represents the degree of correlation between two corresponding variables, and is illustrated by the colour intensity and size of the square, as shown on the scale below the matrix; i.e., small light blue to large dark blue squares represent positive correlation from >0 to +1; small light red to large dark red squares represent negative correlation from <0 to −1. The bacterial taxa included are as follows: Genus1—Unclassified genus within family Ruminococcaceae; Genus2—Unclassified genus within order Bacteroidales; Genus3—Unclassified genus within family Lachnospiraceae; Genus4—Unclassified genus within order Clostridiales. DM—Dry matter (%); GE—Gross energy (KJ/g); NDF—Neutral detergent fibre (%); ADF—Acid detergent fibre (%); NSC—Non-structural carbohydrates (%); HWSC—Hot water soluble carbohydrates (%); Rainfall (mm) and Temperature (°C).

Genus2 (a genus within the phylum Bacteroidetes) was negatively correlated with Genera 1, 3, and 4 (genera within the phylum Firmicutes) (Figure 7). Genus2 was positively correlated with temperature and pasture DM content, and negatively correlated with pasture protein and HWSC content and rainfall. In contrast, Genera 1, 3, and 4 were negatively correlated with pasture DM, NDF, and ADF content and temperature, and positively correlated with pasture protein and HWSC content and rainfall.

## 4. Discussion

The present study investigated the effects of seasonal changes in the nutrient composition of pasture on the diversity of faecal microbiota in horses grazing on typical New Zealand pasture. Currently, there are limited data on the hindgut or faecal microbiota profile of pasture-fed horses, and this study is the third (after our previous New Zealand work [6] and one UK study [24]) to examine the effects of dietary change on the diversity of faecal microbiota in pasture-fed horses. The results of this study showed significant effects of diet, horse, and season on the diversity within the faecal bacterial community, which was dominated by four genera within two phyla—Firmicutes and Bacteroidetes. The faecal bacterial community profile identified in this study was similar to the profile reported in our previous work on pasture-fed horses in New Zealand [7] and in the UK [24], although there was a lack of bacteria belonging to the Fibrobacteres phylum in our study, compared with the UK study which reported up to 18% of the population belonging to this phylum. This may be due to methodological differences between the two studies or reflect differences in pasture composition.

The faecal samples collected, and bacterial sequences obtained in the present study were representative of the year-round population of faecal microbiota, which comprised a rich and diverse bacterial community. The dominant bacterial phyla detected in the present study were consistent with reports from previous studies that used the Illumina next-generation sequencing technique to examine caecal and faecal microbiota in horses [14,58,59]. Some of the bacterial phyla detected were similar to those identified in other studies of horses using various molecular techniques, but the relative abundances reported in those studies varied considerably [8,9,10,23]. This was perhaps due to the variation in the type of horse selected, the type and composition of the diet fed to the horses, the geographical location, inconsistencies in the management practices, and the variation in molecular and bioinformatics analyses used across the studies [4]. Although similarities in results were apparent, caution is warranted in comparing some of the conflicting findings reported in previous studies to the results obtained in the present study.

Kobayashi and co-workers [23] were the first to report a seasonal variation in the diversity of faecal microbiota in horses fed summer versus winter pasture, using conventional microscopy to enumerate the population of the microbiota. However, given that the faecal bacterial community in forage-fed horses is diet specific [7], the underlying differences in diet (grassland pasture comprising of timothy grass offered during summer and woodland pasture comprising of bamboo grass offered during winter), and perhaps its nutrient composition, may have confounded the effect of season on the microbial diversity reported in that study [23].

The significant effects of diet reported in the present study, were in agreement with the findings of our previous work in New Zealand Thoroughbred horses that were also fed forage-only diets [7]. The bacterial diversity was higher when horses were fed exclusively on pasture in both studies when compared to the period when pasture was supplemented with hay or when the horses were fed on a chopped ensiled forage. While there was a temporal effect on the diversity of faecal bacteria within the two diet periods in the present study, the supplementation of hay appeared to dampen the effects of variations in pasture composition. This dampening effect could be associated with the relatively consistent nutrient composition of hay usually harvested at a fixed time during the year when compared to the variation observed in pasture grazed over several months. However, again caution is warranted in extrapolating this finding to other sources and types of hay, because there may be substantial variation in the nutrient composition depending on the type of grass/legume, stage of growth, climate factors, time of harvest, method of processing and storage, which may vary considerably between batches of hay.

Furthermore, Table 1 in the present study showed decreases in %CP, %NSC, %HWSC and DE of hay that was fed over five months after open/barn storage, even though the hay was harvested and processed as a single batch. This finding indicates that storage conditions have a negative effect on the nutritive value of hay, and perhaps, this may be true for other fermented forages preserved under field conditions over extended periods [60,61]. However, a shelf-life study conducted on a batch of chopped ensiled forage (prepared commercially using forages harvested as a single batch, processed via a controlled fermentation technique, and stored anaerobically in polythene-wrapped packaging) showed no change in nutritive value over a storage period of 12 months [62]. This type of an ensiled forage appears to have a more stable nutrient composition when compared to hay or pasture (present study), and also supports a high diversity of faecal microbiota similar to horses grazing on pasture [7].

The variation in pasture composition and the fluctuations in the diversity of faecal microbiota observed in the present study may have implications for grazing management and the preparation of conserved forages for horses susceptible to perturbations of the hindgut microbiota. It may be hypothesised that good quality hay or ensiled forages of a similar nutrient composition could support a relatively consistent population of faecal microbiota by minimising the fluctuations in microbial diversity observed in horses when fed on pasture. Further investigations on the effects of different types of conserved forages on the diversity of the faecal microbiota in horses fed over an extended period are required.

The temporal effects of season and the more subtle effect of the month on the diversity of the bacterial community in faeces reported in the present study appeared to be driven by the variation in the nutrient composition of pasture. When the pasture was growing (vegetative phase, high %CP and low %CHO), Firmicutes dominated the bacterial community, whereas when the pasture was dry (drought-stressed, low %CP and high %CHO), the abundance of Bacteroidetes increased, at the expense of Firmicutes. In particular, the trend for increasing DM content during the months from January to March (drought period), was associated with low pasture growth, which significantly affected the nutrient composition. This spike observed in the %DM was in agreement with previous reports from the region [21,63] and was closely associated with climatic factors (low rainfall and high temperature). The minimal variation in HWSCs reported in the present study may have been biased by the time at which the pasture samples were collected (around mid-day), when the HWSC content is potentially at its lowest in comparison to the early-morning or late-afternoon hours [22,64]. Subtle differences were observed in the bacterial diversity between the months when horses were grazing exclusively on pasture (April, May—winter/limited pasture; November, December—spring/lush pasture and January, February, March—summer/dry pasture) or when limited pasture was supplemented with hay (June, July, August, September, October). This finding was unique and provided evidence in support of the sensitivity of the next-generation sequencing technique to identify these small changes in the diversity of faecal microbial populations [59].

While the main findings of the present study supported the hypothesis that pasture composition influences the diversity of the faecal microbiota of horses, variation was observed between horses within diet periods. Nevertheless, only a few less abundant phyla differed between horses, whereas the most dominant phyla (Firmicutes and Bacteroidetes) were not significantly different. This finding indicated that the group of horses in the study shared a core microbiome, with minimal variation between the horses, and is similar to previous reports on other groups of horses where the diet and management were relatively consistent [8,9,12]. However, the subtle differences in the less abundant bacterial taxa detected between individual horses, even though they were managed as a single cohort under similar conditions, may be associated with the feed intake of individual horses and the retention times of digesta through the gastrointestinal tract. Some horses consume more feed than others, a behaviour that appears to affect the transit time of digesta through the gastrointestinal tract [65,66], and consequently, affects the time available for microbial digestion. This digestive strategy may explain one of the reasons why some horses (due to their inherent feeding behaviour and individual hindgut microbiota) may adapt to dietary changes better than other horses or why some horses may be predisposed to hindgut dysbiosis that induces acute colic or laminitis [13,14]. This further suggests that the population of hindgut/faecal microbiota in some horses is more resilient than in other horses, i.e., the microbial populations of some horses bounce back or adapt better than other horses, although this requires further investigation.

When the %DM of pasture increased, the relative abundance of the phylum Bacteroidetes increased and that of Firmicutes decreased, whilst the remaining phyla of lower relative abundances remained fairly constant. This finding suggests that major shifts in bacterial diversity and abundance can occur in these two phyla, and they should be considered important when monitoring changes in microbial populations. Similar shifts in the Firmicutes:Bacteroidetes ratio have been reported in other studies on the gut microbiome of horses [10,12,59,67] and humans [68,69,70,71] in relation to dietary manipulation. Furthermore, within the phylum Firmicutes, three of the most dominant genera were classified within the order Clostridiales, indicating the importance of this group of bacteria and its association with dietary changes and the development of intestinal dysbiosis [13]. However, the functional roles of these unclassified genera within the order Clostridiales are still unknown and require further investigation.

## 5. Conclusions

The present study showed that the faecal microbiota of pasture-fed horses was highly diverse, and their community structure was diet-specific and associated with the macronutrient composition of the forage. The population structure of the bacterial community, albeit dominated by two bacterial phyla, was dynamic with relative abundances of bacterial genera that fluctuated over time. These fluctuations appeared to be driven by seasonal changes in pasture composition associated in turn with climate factors such as rainfall and temperature. It would be interesting to investigate the resilience of faecal microbiota following dietary alterations, i.e., how do their abundances bounce back after a shift during the dry pasture periods? However, measuring this aspect was beyond the scope of the present study, which only collected snapshot data at monthly intervals for a 12-month period. The outcome of this study suggests future studies can be designed to examine the rate of change in the diversity of faecal microbiota and its resilience following dietary alterations and investigate the functional roles played by the dominant bacterial genera at different times of the year.

## Figures and Tables

**Table 1:** Nutrient composition of pasture and hay and climate variables measured over a period of 12 months.

Nutrient Composition	Diet	Apr	May	Jun	Jul	Aug	Sep	Oct	Nov	Dec	Jan	Feb	Mar
DM (%)	P	21.7	17.6	17.4	17.0	18.5	19.1	23.5	20.2	20.9	26.6	44.6	52.0
	H			94.4	95.7	96.3	96.0	96.3					
CP (%)	P	21.0	24.2	26.9	27.5	24.7	21.9	25.5	21.1	28.2	19.9	15.1	13.2
	H			15.0	9.3	11.1	8.8	9.8					
Fat (%)	P	3.2	3.6	3.8	3.4	3.3	3.6	3.9	3.7	4.4	3.3	2.3	2.1
	H			1.4	1.4	1.4	1.5	1.5					
CHO ^‡^ (%)	P	67.0	58.5	58.1	55.0	54.7	61.1	65.7	70.5	61.3	72.4	77.6	79.7
	H			76.2	82.4	79.2	81.9	80.4					
Ash (%)	P	13.0	17.8	15.3	17.8	20.5	18.0	10.1	9.7	11.2	10.0	10.2	9.2
	H			11.0	8.6	9.1	8.9	9.2					
HWSC (%)	P	8.9	10.6	11.4	10.1	10.1	11.6	13.4	14.7	9.1	8.4	7.5	8.2
	H			9.7	8.8	6.6	7.4	6.4					
NDF (%)	P	52.6	46.2	46.2	46.1	43.7	45.6	43.5	38.7	39.1	52.4	58.0	58.7
	H			43.1	58.8	58.9	62.4	63.4					
ADF (%)	P	28.6	27.6	25.8	26.8	28.7	27.3	22.3	20.6	22.6	27.5	31.3	31.8
	H			30.2	34.6	36.2	36.5	36.8					
ADL (%)	P	2.9	6.4	3.9	6.6	9.5	5.9	0.9	1.0	0.8	0.9	1.1	0.7
	H			0.3	0.5	0.5	0.4	0.5					
NSC ^§^ (%)	P	10.1	8.2	7.8	5.2	7.8	10.8	17.0	26.8	17.1	14.5	14.4	16.8
	H			29.5	21.9	19.5	18.4	16.3					
GE (KJ/g)	P	18.5	17.7	18.3	17.7	16.8	17.3	19.1	19.0	19.3	18.7	18.2	18.3
	H			17.8	17.8	18.1	18.0	17.9					
DE ^†^ (MJ/kg DM)	P	8.1	7.8	8.3	10.0	10.8	10.2	9.2	8.5	8.7	8.5	8.2	8.6
	H			9.8	8.8	8.6	8.3	8.1					
Pasture variables													
Sward height (cm)		3	4	4	4	4	4	5	7	7	6	4	4
Pasture cover (kg DM/ha)		1290	1540	1300	1300	1300	1580	1780	2330	2500	2240	1710	1540
Climate variables													
Mean daily Rainfall (mm)		3.7	1.1	2.8	1.8	1.9	4.6	3.9	2.1	2.2	1.9	0.8	0.4
Mean Temperature (max °C)		20	16	14	14	15	16	18	21	22	22	24	22

P—pasture, H—hay; DM—dry matter; CP—crude protein; CHO—total carbohydrates; HWSC—hot water-soluble carbohydrates; NDF—neutral detergent fibre; ADF—acid detergent fibre; ADL—acid detergent lignin; NSC—non-structural carbohydrates; GE—gross energy; DE—digestible energy. ^†^ Digestible energy (DE) = 2118 + 12.18 (CP) − 9.37 (ADF) − 3.83 (NDF-ADF) + 47.18 (fat) + 20.35 (NSC) − 26.3 (Ash); ^‡^ Total carbohydrates (CHO) = 100 − (CP + fat + ash); ^§^ Non-structural carbohydrates (NSC) = 100 − (CP + fat + ash + NDF).

**Table 2:** Metrics of sequencing and quality screening.

Details	Bacterial Sequences
Initial QC processed reads ^§^	
Total number of reads	16,075,202
Mean number of reads per sample	117,448
(range)	(47,154–189,708)
High quality reads used in downstream analysis ^†^	
Total number of reads	5,257,753
Mean number of reads per sample	44,557
(range)	(19,250–73,405)
Mean length of reads (bp)	356
(range)	(250–486)
Total OTUs detected at 97% similarity	123,645

^†^ After using DynamicTrim, LengthShort, Chimera check; ^§^ after using SolexaQA++, fastaQC, fastQscreen, BWA PhiX, fastq-mcf, flash.

**Table 3:** Comparison of alpha diversity between diets.

Diversity Indices	Pasture		Pasture + Hay		*p*-Value
	Median	IQR	Median	IQR	
Simpson’s (diversity)	0.90	(0.89–0.91)	0.89	(0.88–0.90)	<0.001 *
Shannon-Weiner (entropy)	3.02	(2.93–3.12)	2.91	(2.84–2.96)	<0.001 *
Chao1 (richness)	167	(157–184)	161	(145–179)	0.075

* Level of significance was *p* ≤ 0.05.

**Table 4:** Comparison of Bray-Curtis dissimilarity indices between the horses within each diet period.

Horse	Pasture		Pasture + Hay	
	Median	IQR	Median	IQR
1	0.21	(0.17–0.25)	0.20	(0.17–0.24)
2	0.18	(0.14–0.20)	0.18	(0.15–0.21)
3	0.19	(0.16–0.24)	0.23	(0.19–0.26)
4	0.21	(0.16–0.25)	0.23	(0.18–0.29)
5	0.21	(0.16–0.28)	0.18	(0.16–0.24)
6	0.23	(0.19–0.28)	0.20	(0.18–0.24)
7	0.19	(0.15–0.22)	0.17	(0.14–0.21)
8	0.22	(0.20–0.24)	0.21	(0.16–0.23)
9	0.21	(0.18–0.27)	0.22	(0.21–0.26)
10	0.22	(0.18–0.28)	0.24	(0.19–0.27)
*p* value	<0.007 *		<0.001 *	

* Level of significance was *p* ≤ 0.05 with Steel-Dwass test for multiple comparisons.

**Table 5:** Comparison of the median alpha diversity indices between seasons.

Diversity Indices	Autumn	Winter	Summer	Spring	*p*-Value
Simpson’s (diversity)	0.91 ^b^	0.89 ^a^	0.89 ^a^	0.89 ^a^	0.0001
Shannon-Wiener (entropy)	3.08 ^a^	2.93 ^bc^	3.00 ^a^	2.91 ^c^	0.0001
Chao1 (richness)	173 ^a^	169 ^a^	173 ^a^	154 ^b^	0.0002

Summer—December, January, February; Autumn—March, April, May; Winter—June, July, August; Spring—September, October, November. ^a,b,c^—Different superscripts within a row represent significant differences (*p <* 0.005).

**Table 6:** Comparison of the median alpha diversity indices between months.

Diversity Indices	Apr	May	Jun	Jul	Aug	Sep	Oct	Nov	Dec	Jan	Feb	Mar	*p*-Value
Simpson’s (diversity)	0.90	0.90	0.90	0.89	0.88	0.89	0.89	0.90	0.89	0.89	0.92	0.92	0.0001
Shannon-Wiener (entropy)	3.03	2.99	2.95	2.92	2.90	2.89	2.90	2.94	2.93	2.97	3.19	3.14	0.0001
Chao1 (richness)	162	169	171	187	142	148	165	144	162	162	192	181	0.0001

## Data Availability

The project is registered with NCBI PRJNA286058, and the sequence data generated in this study are available via the Sequence Read Archive under the accession number SRA272143.

## Supplementary Materials

The following are available online at https://www.mdpi.com/article/10.3390/ani11082300/s1, Text S1: Detailed materials and methods, Figure S1: Principal coordinate analysis of the faecal bacterial community by season and month, Figure S2: Season-wise hierarchial clustering of the faecal bacterial community of horses included in the study, Table S1: Metadata on the faecal samples (n = 118) included in the study on the seasonal variation in the faecal microbiota of pasture-fed horses (n = 10), Table S2: Comparison of mean relative abundance at phylum level between diet periods, Table S3: Comparison of the mean relative abundance at genus level between diet periods, Table S4: Comparison of mean relative abundance at phylum level between horses, Table S5: Comparison of the mean relative abundance at genus level between horses, Table S6: Comparison of mean relative abundance at phylum level between seasons, Table S7: Comparison of mean relative abundance at genus level between seasons, Table S8: Comparison of mean relative abundance at phylum level between months, Table S9: Comparison of mean relative abundance at genus level between months.
