# Supplementary material for: Seasonal Variation in the Faecal Microbiota of Mature Adult Horses Maintained on Pasture in New Zealand
Source: Animals (Basel). 2021 Aug 4;11(8):2300. doi: 10.3390/ani11082300 (PMC8388417; doi:10.3390/ani11082300)

Text S1. Detailed materials and methods

DNA extraction method

The cells were disrupted by bead-beating for 4 min at 2100 oscillations s^{-1} (Mini-Beadbeater-96, BioSpec, Bartlesville, OK, USA), with 0.7 g Zirconia beads (0.1 mm; dnature, Gisborne, New Zealand), 550 µl phenol-chloroform-isoamylalcohol (25:24:1 vol:vol:vol; pH 8), 282 µl buffer A (200 mM NaCl, 200 mM Tria, 20 mM EDTA, pH 8 with NaOH), 268 µl PM buffer (Qiagen, Hilden, Germany), and 200 µl sodium dodecyl sulphate (20% wt/vol) [1,2]. After centrifugation at 20,000 x *g* at 4°C for 20 min, 350 µl of the supernatant was removed, and treated at 37°C for 15 min with 5 µl RNase A (Life Technologies, Thermo Fisher Scientific, Auckland, New Zealand) [3]. The supernatant was then mixed with 650 µl PM buffer (Qiagen) and processed through the QIAquick column by applying a vacuum with a QIAvac 96 vacuum manifold (Qiagen). The membrane-bound DNA was washed with 750 µl PE buffer (Qiagen) twice as recommended by the manufacturer, and DNA was eluted in 80 µl elution buffer (10 mM Tris; pH 8.5 with HCl) and stored at -80°C.

PCR amplification and sequencing protocol

Primer and adapter sequences

To amplify and sequence the V3-V4 hypervariable region of the 16S rRNA gene, specific bacterial primer sequences were selected, S-D-Bact-0341-b-S-17 and S-D-Bact-0785-a-A-21 [4]. The full length primer sequences, using standard IUPAC nucleotide nomenclature, were as follows:

16S Amplicon PCR Forward Primer:

5'TCGTCGGCAGCGTCAGATGTGTATAAGAGACAGCCTACGGGNGGCWGCAG

16S Amplicon PCR Reverse Primer:

5'GTCTCGTGGGCTCGGAGATGTGTATAAGAGACAGGACTACHVGGGTATCTAATCC

The Illumina overhang adapter sequences added to the locus-specific sequences were:

Forward overhang: 5' TCGTCGGCAGCGTCAGATGTGTATAAGAGACAG-[locus-specific sequence]

Reverse overhang: 5' GTCTCGTGGGCTCGGAGATGTGTATAAGAGACAG-[locus-specific sequence]

Amplicon PCR

The amplicons were prepared using 2.5 µl gDNA (5 ng/ µl), 5 µl forward primer (1 µM), 5 µl reverse primer (1 µM), 12.5 µl 2x KAPA HiFi HotStart Ready Mix, to make a master mix reaction of 25 µl. PCR was performed on a Thermocycler ProS (Eppendorf, Hamburg, Germany) using the following program: initial denaturation at 95°C for 3 minutes, 25 cycles of denaturation at 95°C for 30 seconds, annealing at 55°C for 30 seconds and elongation at 72°C for 30 seconds, followed by a final elongation

at 72°C for 5 minutes and held at 4°C. The amplicons were then purified using AMPure XP beads (AMPure, Agencourt, Beckman Coulter, Beverly, MA, USA).

Index PCR

The linker primer sequence used for the samples was GTGCCAGCMGCCGCGGTAA and the 8 bp unique barcodes are given in Table S1. The dual indices and Illumina sequencing adapters were attached to the PCR amplicons by the following protocol: 5 µl gDNA, 5 µl Nextera XT Index Primer 1 (N7xx), 5 µl Nextera XT Index Primer 2 (S5xx), 25 µl 2x KAPA HiFi HotStart Ready Mix, 10 µl PCR grade water, to make a total master mix of 50 µl. PCR was performed on a Thermocycler ProS (Eppendorf) using the following program: initial denaturation at 95°C for 3 minutes, 8 cycles of denaturation at 95°C for 30 seconds, annealing at 55°C for 30 seconds and elongation at 72°C for 30 seconds, followed by a final elongation at 72°C for 5 minutes and held at 4°C. The amplicons were then purified using AMPure XP beads (AMPure) [5].

Library validation

A sample of 1 µl of a 1:50 dilution of the final library was run on a Bioanalyzer DNA 1000 chip (2100 Bioanalyzer (Agilent Technologies, Santa Clara, CA, USA) to verify the size of the amplicons, which was expected to be ~630 bp when using the V3 and V4 primer pairs in the protocol.

References

1. Rius A, Kittelmann S, Macdonald K, Waghorn G, Janssen P, et al. (2012) Nitrogen metabolism and rumen microbial enumeration in lactating cows with divergent residual feed intake fed high-digestibility pasture. Journal of dairy science 95: 5024-5034.
2. Kittelmann S, Pinares-Patiño CS, Seedorf H, Kirk MR, Ganesh S, et al. (2014) Two Different Bacterial Community Types Are Linked with the Low-Methane Emission Trait in Sheep. PLoS ONE 9: e103171.
3. Healey A, Furtado A, Cooper T, Henry R (2014) Protocol: a simple method for extracting next-generation sequencing quality genomic DNA from recalcitrant plant species. Plant Methods 10: 21.
4. Klindworth A, Pruesse E, Schweer T, Peplies J, Quast C, et al. (2012) Evaluation of general 16S ribosomal RNA gene PCR primers for classical and next-generation sequencing-based diversity studies. Nucleic acids research: gks808.
5. Fadrosh DW, Ma B, Gajer P, Sengamalay N, Ott S, et al. (2014) An improved dual-indexing approach for multiplexed 16S rRNA gene sequencing on the Illumina MiSeq platform. Microbiome 2: 1-7.

Table S1. Metadata on the faecal samples (n=118) included in the study on the seasonal variation in the faecal microbiota of pasture-fed horses (n=10)

SampleID	BarcodeSequenceID	HorseID	Breed	AgeYears	Month	Season	Diet	Location
Jan_H1	TAAGGCGA	H1	Standardbred	26	January	Summer	Pasture	Paddock_3
Jan_H2	CGTACTAG	H2	Thoroughbred	15	January	Summer	Pasture	Paddock_3
Jan_H3	AGGCAGAA	H3	Standardbred	9	January	Summer	Pasture	Paddock_3
Jan_H4	TCCTGAGC	H4	Standardbred	19	January	Summer	Pasture	Paddock_3
Jan_H5	GGACTCCT	H5	Thoroughbred	12	January	Summer	Pasture	Paddock_3
Jan_H6	TAGGCATG	H6	Standardbred	17	January	Summer	Pasture	Paddock_3
Jan_H7	CTCTCTAC	H7	Standardbred	8	January	Summer	Pasture	Paddock_3
Jan_H8	CAGAGAGG	H8	Standardbred	12	January	Summer	Pasture	Paddock_3
Jan_H9	GCTACGCT	H9	Standardbred	19	January	Summer	Pasture	Paddock_3
Jan_H10	CGAGGCTG	H10	Standardbred	14	January	Summer	Pasture	Paddock_3
Feb_H1	AAGAGGCA	H1	Standardbred	26	February	Summer	Pasture	Paddock_3
Feb_H2	GTAGAGGA	H2	Thoroughbred	15	February	Summer	Pasture	Paddock_3
Feb_H3	TAAGGCGA	H3	Standardbred	9	February	Summer	Pasture	Paddock_3
Feb_H4	CGTACTAG	H4	Standardbred	19	February	Summer	Pasture	Paddock_3
Feb_H5	AGGCAGAA	H5	Thoroughbred	12	February	Summer	Pasture	Paddock_3
Feb_H6	TCCTGAGC	H6	Standardbred	17	February	Summer	Pasture	Paddock_3
Feb_H7	GGACTCCT	H7	Standardbred	8	February	Summer	Pasture	Paddock_3
Feb_H8	TAGGCATG	H8	Standardbred	12	February	Summer	Pasture	Paddock_3
Feb_H9	CTCTCTAC	H9	Standardbred	19	February	Summer	Pasture	Paddock_3
Feb_H10	CAGAGAGG	H10	Standardbred	14	February	Summer	Pasture	Paddock_3
Mar_H1	GCTACGCT	H1	Standardbred	26	March	Autumn	Pasture	Paddock_3
Mar_H2	CGAGGCTG	H2	Thoroughbred	15	March	Autumn	Pasture	Paddock_3
Mar_H3	AAGAGGCA	H3	Standardbred	9	March	Autumn	Pasture	Paddock_3
Mar_H4	GTAGAGGA	H4	Standardbred	19	March	Autumn	Pasture	Paddock_3
Mar_H5	TAAGGCGA	H5	Thoroughbred	12	March	Autumn	Pasture	Paddock_3
Mar_H6	CGTACTAG	H6	Standardbred	17	March	Autumn	Pasture	Paddock_3
Mar_H7	AGGCAGAA	H7	Standardbred	8	March	Autumn	Pasture	Paddock_3
Mar_H8	TCCTGAGC	H8	Standardbred	12	March	Autumn	Pasture	Paddock_3
Mar_H9	GGACTCCT	H9	Standardbred	19	March	Autumn	Pasture	Paddock_3

Mar_H10	TAGGCATG	H10	Standardbred	14	March	Autumn	Pasture	Paddock_3
Apr_H1	CTCTCTAC	H1	Standardbred	26	April	Autumn	Pasture	Paddock_1
Apr_H2	CAGAGAGG	H2	Thoroughbred	15	April	Autumn	Pasture	Paddock_1
Apr_H3	GCTACGCT	H3	Standardbred	9	April	Autumn	Pasture	Paddock_1
Apr_H4	CGAGGCTG	H4	Standardbred	19	April	Autumn	Pasture	Paddock_1
Apr_H5	AAGAGGCA	H5	Thoroughbred	12	April	Autumn	Pasture	Paddock_1
Apr_H6	GTAGAGGA	H6	Standardbred	17	April	Autumn	Pasture	Paddock_1
Apr_H7	TAAGGCGA	H7	Standardbred	8	April	Autumn	Pasture	Paddock_1
Apr_H8	CGTACTAG	H8	Standardbred	12	April	Autumn	Pasture	Paddock_1
Apr_H9	AGGCAGAA	H9	Standardbred	19	April	Autumn	Pasture	Paddock_1
Apr_H10	TCCTGAGC	H10	Standardbred	14	April	Autumn	Pasture	Paddock_1
May_H1	GGACTCCT	H1	Standardbred	26	May	Autumn	Pasture	Paddock_1
May_H2	TAGGCATG	H2	Thoroughbred	15	May	Autumn	Pasture	Paddock_1
May_H3	CTCTCTAC	H3	Standardbred	9	May	Autumn	Pasture	Paddock_1
May_H4	CAGAGAGG	H4	Standardbred	19	May	Autumn	Pasture	Paddock_1
May_H5	GCTACGCT	H5	Thoroughbred	12	May	Autumn	Pasture	Paddock_1
May_H6	CGAGGCTG	H6	Standardbred	17	May	Autumn	Pasture	Paddock_1
May_H7	AAGAGGCA	H7	Standardbred	8	May	Autumn	Pasture	Paddock_1
May_H8	GTAGAGGA	H8	Standardbred	12	May	Autumn	Pasture	Paddock_1
May_H10	CGTACTAG	H10	Standardbred	14	May	Autumn	Pasture	Paddock_1
Jun_H1	AGGCAGAA	H1	Standardbred	26	June	Winter	Pasture+Hay	Paddock_1
Jun_H2	TCCTGAGC	H2	Thoroughbred	15	June	Winter	Pasture+Hay	Paddock_1
Jun_H3	GGACTCCT	H3	Standardbred	9	June	Winter	Pasture+Hay	Paddock_1
Jun_H4	TAGGCATG	H4	Standardbred	19	June	Winter	Pasture+Hay	Paddock_1
Jun_H5	CTCTCTAC	H5	Thoroughbred	12	June	Winter	Pasture+Hay	Paddock_1
Jun_H6	CAGAGAGG	H6	Standardbred	17	June	Winter	Pasture+Hay	Paddock_1
Jun_H7	GCTACGCT	H7	Standardbred	8	June	Winter	Pasture+Hay	Paddock_1
Jun_H8	CGAGGCTG	H8	Standardbred	12	June	Winter	Pasture+Hay	Paddock_1
Jun_H9	AAGAGGCA	H9	Standardbred	19	June	Winter	Pasture+Hay	Paddock_1
Jun_H10	GTAGAGGA	H10	Standardbred	14	June	Winter	Pasture+Hay	Paddock_1
Jul_H1	TAAGGCGA	H1	Standardbred	26	July	Winter	Pasture+Hay	Paddock_1
Jul_H2	CGTACTAG	H2	Thoroughbred	15	July	Winter	Pasture+Hay	Paddock_1
Jul_H3	AGGCAGAA	H3	Standardbred	9	July	Winter	Pasture+Hay	Paddock_1
Jul_H4	TCCTGAGC	H4	Standardbred	19	July	Winter	Pasture+Hay	Paddock_1

Jul_H5	GGACTCCT	H5	Thoroughbred	12	July	Winter	Pasture+Hay	Paddock_1
Jul_H6	TAGGCATG	H6	Standardbred	17	July	Winter	Pasture+Hay	Paddock_1
Jul_H7	CTCTCTAC	H7	Standardbred	8	July	Winter	Pasture+Hay	Paddock_1
Jul_H8	CAGAGAGG	H8	Standardbred	12	July	Winter	Pasture+Hay	Paddock_1
Jul_H9	GCTACGCT	H9	Standardbred	19	July	Winter	Pasture+Hay	Paddock_1
Jul_H10	CGAGGCTG	H10	Standardbred	14	July	Winter	Pasture+Hay	Paddock_1
Aug_H1	AAGAGGCA	H1	Standardbred	26	August	Winter	Pasture+Hay	Paddock_1
Aug_H2	GTAGAGGA	H2	Thoroughbred	15	August	Winter	Pasture+Hay	Paddock_1
Aug_H3	TAAGGCGA	H3	Standardbred	9	August	Winter	Pasture+Hay	Paddock_1
Aug_H4	CGTACTAG	H4	Standardbred	19	August	Winter	Pasture+Hay	Paddock_1
Aug_H5	AGGCAGAA	H5	Thoroughbred	12	August	Winter	Pasture+Hay	Paddock_1
Aug_H7	GGACTCCT	H7	Standardbred	8	August	Winter	Pasture+Hay	Paddock_1
Aug_H8	TAGGCATG	H8	Standardbred	12	August	Winter	Pasture+Hay	Paddock_1
Aug_H9	CTCTCTAC	H9	Standardbred	19	August	Winter	Pasture+Hay	Paddock_1
Aug_H10	CAGAGAGG	H10	Standardbred	14	August	Winter	Pasture+Hay	Paddock_1
Sept_H1	GCTACGCT	H1	Standardbred	26	September	Spring	Pasture+Hay	Paddock_1
Sept_H2	CGAGGCTG	H2	Thoroughbred	15	September	Spring	Pasture+Hay	Paddock_1
Sept_H3	AAGAGGCA	H3	Standardbred	9	September	Spring	Pasture+Hay	Paddock_1
Sept_H4	GTAGAGGA	H4	Standardbred	19	September	Spring	Pasture+Hay	Paddock_1
Sept_H5	TAAGGCGA	H5	Thoroughbred	12	September	Spring	Pasture+Hay	Paddock_1
Sept_H6	CGTACTAG	H6	Standardbred	17	September	Spring	Pasture+Hay	Paddock_1
Sept_H7	AGGCAGAA	H7	Standardbred	8	September	Spring	Pasture+Hay	Paddock_1
Sept_H8	TCCTGAGC	H8	Standardbred	12	September	Spring	Pasture+Hay	Paddock_1
Sept_H9	GGACTCCT	H9	Standardbred	19	September	Spring	Pasture+Hay	Paddock_1
Sept_H10	TAGGCATG	H10	Standardbred	14	September	Spring	Pasture+Hay	Paddock_1
Oct_H1	CTCTCTAC	H1	Standardbred	26	October	Spring	Pasture+Hay	Paddock_1
Oct_H2	CAGAGAGG	H2	Thoroughbred	15	October	Spring	Pasture+Hay	Paddock_1
Oct_H3	GCTACGCT	H3	Standardbred	9	October	Spring	Pasture+Hay	Paddock_1
Oct_H4	CGAGGCTG	H4	Standardbred	19	October	Spring	Pasture+Hay	Paddock_1
Oct_H5	AAGAGGCA	H5	Thoroughbred	12	October	Spring	Pasture+Hay	Paddock_1
Oct_H6	GTAGAGGA	H6	Standardbred	17	October	Spring	Pasture+Hay	Paddock_1
Oct_H7	TAAGGCGA	H7	Standardbred	8	October	Spring	Pasture+Hay	Paddock_1
Oct_H8	CGTACTAG	H8	Standardbred	12	October	Spring	Pasture+Hay	Paddock_1
Oct_H9	AGGCAGAA	H9	Standardbred	19	October	Spring	Pasture+Hay	Paddock_1

Oct_H10	TCCTGAGC	H10	Standardbred	14	October	Spring	Pasture+Hay	Paddock_1
Nov_H1	GGACTCCT	H1	Standardbred	26	November	Spring	Pasture	Paddock_1
Nov_H2	TAGGCATG	H2	Thoroughbred	15	November	Spring	Pasture	Paddock_1
Nov_H3	CTCTCTAC	H3	Standardbred	9	November	Spring	Pasture	Paddock_1
Nov_H4	CAGAGAGG	H4	Standardbred	19	November	Spring	Pasture	Paddock_1
Nov_H5	GCTACGCT	H5	Thoroughbred	12	November	Spring	Pasture	Paddock_1
Nov_H6	CGAGGCTG	H6	Standardbred	17	November	Spring	Pasture	Paddock_1
Nov_H7	AAGAGGCA	H7	Standardbred	8	November	Spring	Pasture	Paddock_1
Nov_H8	GTAGAGGA	H8	Standardbred	12	November	Spring	Pasture	Paddock_1
Nov_H9	TAAGGCGA	H9	Standardbred	19	November	Spring	Pasture	Paddock_1
Nov_H10	CGTACTAG	H10	Standardbred	14	November	Spring	Pasture	Paddock_1
Dec_H1	AGGCAGAA	H1	Standardbred	26	December	Summer	Pasture	Paddock_2
Dec_H2	TCCTGAGC	H2	Thoroughbred	15	December	Summer	Pasture	Paddock_2
Dec_H3	GGACTCCT	H3	Standardbred	9	December	Summer	Pasture	Paddock_2
Dec_H4	TAGGCATG	H4	Standardbred	19	December	Summer	Pasture	Paddock_2
Dec_H5	CTCTCTAC	H5	Thoroughbred	12	December	Summer	Pasture	Paddock_2
Dec_H6	CAGAGAGG	H6	Standardbred	17	December	Summer	Pasture	Paddock_2
Dec_H7	GCTACGCT	H7	Standardbred	8	December	Summer	Pasture	Paddock_2
Dec_H8	CGAGGCTG	H8	Standardbred	12	December	Summer	Pasture	Paddock_2
Dec_H9	AAGAGGCA	H9	Standardbred	19	December	Summer	Pasture	Paddock_2
Dec_H10	GTAGAGGA	H10	Standardbred	14	December	Summer	Pasture	Paddock_2

Table S2. Comparison of mean relative abundance at phylum level between diet periods.

Taxon	Pasture	Pasture + Hay	P value
Firmicutes	0.611	0.680	<0.001*
Bacteroidetes	0.237	0.194	0.002*
Verrucomicrobia	0.039	0.033	0.003*
Unassigned phyla	0.033	0.023	<0.001*
Actinobacteria	0.018	0.015	0.024
TM7	0.017	0.017	0.737
Spirochaetes	0.016	0.010	0.002*
Proteobacteria	0.006	0.005	<0.001*
Tenericutes	0.006	0.005	0.178
Cyanobacteria	0.004	0.003	0.003*
Fibrobacteres	0.003	0.003	0.591
Euryarchaeota	0.002	0.001	0.005
WPS-2	0.001	0.002	0.119
Fusobacteria	<0.001	<0.001	0.745
Other Phyla <1%	0.007	0.007	0.336

*Level of significance was $P \leq 0.003$ after Bonferroni correction for multiple comparisons

Table S3. Comparison of the mean relative abundance at genus level between diet periods.

Taxon	Pasture	Pasture+Hay	P value
Firmicutes>Clostridia>Clostridiales>Ruminococcaceae>unclassified genus	0.206	0.234	<0.001*
Firmicutes>Clostridia>Clostridiales>Lachnospiraceae>unclassified genus	0.135	0.136	0.581
Firmicutes>Clostridia>Clostridiales>unclassified family>unclassified genus	0.116	0.140	<0.001*
Bacteroidetes>Bacteroidia>Bacteroidales>unclassified family>unclassified genus	0.108	0.086	0.002
Unassigned genera	0.033	0.023	<0.001*
Verrucomicrobia>Verruco-5>WCHB1-41>RFP12>unclassified genus	0.023	0.019	<0.001*
Bacteroidetes>Bacteroidia>Bacteroidales>Prevotellaceae>Prevotella	0.023	0.020	0.132
Firmicutes>Clostridia>Clostridiales>[Mogibacteriaceae]>unclassified genus	0.021	0.024	0.042
Firmicutes>Clostridia>Clostridiales>Ruminococcaceae>Ruminococcus	0.021	0.023	0.366
Bacteroidetes>Bacteroidia>Bacteroidales>BS11>unclassified genus	0.019	0.021	0.920
Bacteroidetes>Bacteroidia>Bacteroidales>[Paraprevotellaceae]>unclassified genus	0.018	0.020	0.876
TM7>TM7-3>CW040>F16>unclassified genus	0.017	0.017	0.702
Bacteroidetes>Bacteroidia>Bacteroidales>[Paraprevotellaceae]>YRC22	0.015	0.015	0.668
Verrucomicrobia>Verrucomicrobiae>Verrucomicrobiales>Verrucomicrobiaceae>Akkermansia	0.014	0.014	0.058
Bacteroidetes>Bacteroidia>Bacteroidales>[Paraprevotellaceae]>CF231	0.013	0.007	<0.001*
Spirochaetes>Spirochaetes>Spirochaetales>Spirochaetaceae>Treponema	0.012	0.009	0.011
Firmicutes>Clostridia>Clostridiales>Lachnospiraceae>Coprococcus	0.011	0.013	0.046
Firmicutes>Erysipelotrichi>Erysipelotrichales>Erysipelotrichaceae>p-75-a5	0.011	0.012	0.310
Firmicutes>Clostridia>Clostridiales>Veillonellaceae>Phascolarctobacterium	0.009	0.006	0.005
Bacteroidetes>Bacteroidia>Bacteroidales>RF16>unclassified genus	0.008	0.003	<0.001*
Bacteroidetes>Bacteroidia>Bacteroidales>Bacteroidaceae>BF311	0.008	0.007	0.036
Firmicutes>Clostridia>Clostridiales>Ruminococcaceae>Oscillospira	0.008	0.009	0.307
Bacteroidetes>Bacteroidia>Bacteroidales>Porphyromonadaceae>Paludibacter	0.008	0.002	0.036
Firmicutes>Clostridia>Clostridiales>Clostridiaceae>Clostridium	0.007	0.008	0.544
Firmicutes>Clostridia>Clostridiales>Clostridiaceae>unclassified genus	0.007	0.013	<0.001*
Actinobacteria>Coriobacteriia>Coriobacteriales>Coriobacteriaceae>unclassified genus	0.007	0.008	0.063
Firmicutes>Clostridia>Clostridiales>Lachnospiraceae;Other	0.006	0.005	0.036
Firmicutes>Clostridia>Clostridiales>Christensenellaceae>unclassified genus	0.006	0.008	<0.001*

Bacteroidetes>Bacteroidia>Bacteroidales>[Paraprevotellaceae]>[Prevotella]	0.005	0.004	0.019
Firmicutes>Clostridia>Clostridiales>Lachnospiraceae>Blautia	0.005	0.004	0.288
Firmicutes>Clostridia>Clostridiales>Lachnospiraceae>Pseudobutyrivibrio	0.004	0.004	0.221
Firmicutes>Clostridia>Clostridiales>Eubacteriaceae>Pseudoramibacter_Eubacterium	0.004	0.005	0.881
Firmicutes>Bacilli>Lactobacillales>Lactobacillaceae>Lactobacillus	0.004	0.008	<0.001*
Tenericutes>Mollicutes>RF39>unclassified family>unclassified genus	0.004	0.005	0.387
Bacteroidetes>Bacteroidia>Bacteroidales>Bacteroidaceae>Bacteroides	0.004	0.004	0.003
Firmicutes>Clostridia>Clostridiales>unclassified family>unclassified genus	0.004	0.003	0.044
Cyanobacteria>4C0d-2>YS2>unclassified family>unclassified genus	0.004	0.003	0.004
Fibrobacteres>Fibrobacteria>Fibrobacterales>Fibrobacteraceae>Fibrobacter	0.003	0.003	0.591
Spirochaetes>MVP-15>PL-11B10>unclassified family>unclassified genus	0.003	0.001	0.864
Actinobacteria>Actinobacteria>Actinomycetales>Nocardiaceae>Rhodococcus	0.002	0.001	<0.001*
Firmicutes>Erysipelotrichi>Erysipelotrichales>Erysipelotrichaceae>RFN20	0.002	0.001	0.031
Bacteroidetes>Bacteroidia>Bacteroidales>Bacteroidaceae>unclassified genus	0.002	0.002	0.128
Firmicutes>Clostridia>Clostridiales>Veillonellaceae>unclassified genus	0.002	0.004	0.022
WPS-2>unclassified class>unclassified order>unclassified family>unclassified genus	0.001	0.002	0.119
Firmicutes>Clostridia>Clostridiales>Clostridiaceae>Sarcina	0.001	0.003	<0.001*
Firmicutes>Clostridia>Clostridiales>Lachnospiraceae>[Ruminococcus]	0.001	0.003	0.484
Euryarchaeota>Methanobacteria>Methanobacteriales>Methanobacteriaceae>Methanobrevibacter	0.001	0.001	0.741
Verrucomicrobia>Verruco-5>LD1-PB3>unclassified family>unclassified genus	<0.001	<0.001	0.549
Firmicutes>Clostridia>Clostridiales>Lachnospiraceae>Epulopiscium	<0.001	0.001	0.015
Proteobacteria>Gammaproteobacteria>Enterobacteriales>Enterobacteriaceae>unclassified genus	<0.001	<0.001	0.049
Fusobacteria>Fusobacteriia>Fusobacteriales>Fusobacteriaceae>Fusobacterium	<0.001	<0.001	0.545
Other Genera <1%	0.049	0.039	<0.001*

*Level of significance was P≤0.001 after Bonferroni correction for multiple comparisons

Table S4. Comparison of mean relative abundance at phylum level between horses.

Taxon	H1	H2	H3	H4	H5	H6	H7	H8	H9	H10	P value
Firmicutes	0.608	0.655	0.605	0.635	0.634	0.656	0.648	0.655	0.620	0.680	0.281
Bacteroidetes	0.239	0.224	0.24	0.225	0.231	0.195	0.221	0.203	0.232	0.178	0.436
Unassigned phyla	0.033	0.028	0.032	0.027	0.033	0.026	0.025	0.026	0.029	0.030	0.199
Verrucomicrobia	0.032	0.027	0.036	0.040	0.029	0.04	0.027	0.04	0.045	0.053	0.001*
TM7	0.025	0.009	0.024	0.019	0.017	0.018	0.015	0.022	0.01	0.012	0.007
Spirochaetes	0.017	0.018	0.015	0.011	0.014	0.010	0.015	0.011	0.017	0.009	0.049
Actinobacteria	0.016	0.014	0.017	0.015	0.015	0.023	0.020	0.016	0.018	0.017	0.359
Tenericutes	0.006	0.005	0.008	0.006	0.005	0.006	0.006	0.006	0.005	0.004	0.002*
Proteobacteria	0.005	0.005	0.005	0.005	0.005	0.009	0.006	0.006	0.006	0.004	0.180
Cyanobacteria	0.005	0.004	0.004	0.003	0.004	0.003	0.003	0.004	0.003	0.002	0.009
Fibrobacteres	0.004	0.003	0.004	0.003	0.005	0.003	0.003	0.001	0.004	0.002	0.098
Euryarchaeota	0.002	0.001	0.002	0.001	0.001	0.002	0.002	0.001	0.001	0.001	0.088
WPS-2	0.002	0.001	0.002	0.001	0.001	0.004	0.002	0.002	0.002	0.001	0.049
Fusobacteria	<0.001	<0.001	<0.001	0.001	<0.001	<0.001	<0.001	<0.001	<0.001	<0.001	0.826
Other Phyla <1%	0.007	0.005	0.007	0.008	0.007	0.006	0.006	0.007	0.01	0.008	0.001*

*Level of significance was P≤0.003 after Bonferroni correction for multiple comparisons

Table S5. Comparison of the mean relative abundance at genus level between horses.

Taxon	H1	H2	H3	H4	H5	H6	H7	H8	H9	H10	P value
Firmicutes>Clostridia>Clostridiales>Ruminococcaceae>unclassified genus	0.203	0.221	0.218	0.210	0.223	0.221	0.220	0.243	0.207	0.208	0.450
Firmicutes>Clostridia>Clostridiales>Lachnospiraceae>unclassified genus	0.140	0.163	0.104	0.141	0.122	0.145	0.127	0.120	0.140	0.153	0.002
Firmicutes>Clostridia>Clostridiales>unclassified family>unclassified genus	0.119	0.116	0.128	0.125	0.129	0.129	0.127	0.129	0.117	0.14	0.737
Bacteroidetes>Bacteroidia>Bacteroidales>unclassified family>unclassified genus	0.114	0.094	0.110	0.098	0.105	0.091	0.109	0.084	0.110	0.077	0.259
Unassigned genera	0.033	0.028	0.032	0.027	0.033	0.026	0.025	0.026	0.029	0.030	0.199
TM7>TM7-3>CW040>F16>unclassified genus	0.025	0.009	0.024	0.018	0.016	0.018	0.015	0.022	0.010	0.012	0.007
Firmicutes>Clostridia>Clostridiales>[Mogibacteriaceae]>unclassified genus	0.023	0.016	0.023	0.021	0.023	0.020	0.020	0.024	0.025	0.027	0.126
Bacteroidetes>Bacteroidia>Bacteroidales>BS11>unclassified genus	0.022	0.017	0.026	0.021	0.020	0.018	0.013	0.027	0.017	0.018	0.097
Bacteroidetes>Bacteroidia>Bacteroidales>[Paraprevotellaceae]>YRC22	0.022	0.014	0.013	0.014	0.016	0.013	0.014	0.011	0.023	0.011	0.157
Verrucomicrobia>Verruco-5>WCHB1-41>RFP12>unclassified genus	0.020	0.021	0.023	0.025	0.017	0.021	0.016	0.017	0.028	0.026	0.004
Firmicutes>Clostridia>Clostridiales>Ruminococcaceae>Ruminococcus	0.020	0.025	0.021	0.021	0.026	0.018	0.026	0.020	0.017	0.021	0.348
Bacteroidetes>Bacteroidia>Bacteroidales>[Paraprevotellaceae]>unclassified genus	0.016	0.016	0.023	0.016	0.025	0.015	0.015	0.022	0.018	0.023	0.057
Bacteroidetes>Bacteroidia>Bacteroidales>Prevotellaceae>Prevotella	0.014	0.027	0.020	0.023	0.026	0.017	0.023	0.028	0.022	0.016	0.135
Bacteroidetes>Bacteroidia>Bacteroidales>Bacteroidaceae>BF311	0.014	0.005	0.009	0.008	0.006	0.008	0.010	0.005	0.004	0.006	0.005
Bacteroidetes>Bacteroidia>Bacteroidales>[Paraprevotellaceae]>CF231	0.012	0.018	0.009	0.011	0.010	0.010	0.013	0.006	0.011	0.007	0.022
Spirochaetes>Spirochaetes>Spirochaetales>Spirochaetaceae>Treponema	0.011	0.016	0.011	0.009	0.009	0.009	0.011	0.008	0.014	0.008	0.009
Verrucomicrobia>Verrucomicrobiae>Verrucomicrobiales>Verrucomicrobiaceae>Akkermansia	0.011	0.005	0.012	0.014	0.011	0.019	0.010	0.020	0.016	0.026	0.002
Firmicutes>Clostridia>Clostridiales>Lachnospiraceae>Coprococcus	0.010	0.011	0.008	0.010	0.012	0.012	0.016	0.011	0.012	0.016	0.035
Firmicutes>Clostridia>Clostridiales>Clostridiaceae>unclassified genus	0.009	0.005	0.007	0.011	0.010	0.011	0.007	0.011	0.012	0.013	0.017
Firmicutes>Clostridia>Clostridiales>Ruminococcaceae>Oscillospira	0.008	0.011	0.008	0.008	0.008	0.007	0.009	0.010	0.007	0.008	0.245
Firmicutes>Erysipelotrichi>Erysipelotrichales>Erysipelotrichaceae>p-75-a5	0.008	0.013	0.013	0.010	0.009	0.015	0.010	0.015	0.009	0.009	0.077
Actinobacteria>Coriobacteriia>Coriobacteriales>Coriobacteriaceae>unclassified genus	0.007	0.006	0.007	0.007	0.007	0.009	0.009	0.008	0.009	0.009	0.463
Firmicutes>Clostridia>Clostridiales>Christensenellaceae>unclassified genus	0.006	0.005	0.008	0.006	0.007	0.006	0.008	0.008	0.006	0.006	0.019
Bacteroidetes>Bacteroidia>Bacteroidales>[Paraprevotellaceae]>[Prevotella]	0.006	0.009	0.002	0.006	0.004	0.004	0.004	0.002	0.005	0.006	0.002
Bacteroidetes>Bacteroidia>Bacteroidales>RF16>unclassified genus	0.006	0.006	0.007	0.008	0.005	0.008	0.005	0.004	0.007	0.005	0.967
Firmicutes>Clostridia>Clostridiales>Clostridiaceae>Clostridium	0.006	0.008	0.006	0.009	0.008	0.005	0.009	0.007	0.008	0.008	0.035
Firmicutes>Clostridia>Clostridiales>Veillonellaceae>Phascolarctobacterium	0.005	0.011	0.005	0.007	0.011	0.005	0.009	0.005	0.009	0.010	0.031
Tenericutes>Mollicutes>RF39>unclassified family>unclassified genus	0.005	0.004	0.006	0.005	0.004	0.005	0.004	0.005	0.003	0.003	0.004
Firmicutes>Clostridia>Clostridiales>Veillonellaceae>unclassified genus	0.005	0.003	0.003	0.003	0.003	0.001	0.002	0.003	0.003	0.002	0.234

Firmicutes>Clostridia>Clostridiales>Lachnospiraceae>unclassified genus	0.005	0.008	0.005	0.007	0.005	0.007	0.007	0.004	0.004	0.004	0.003
Cyanobacteria>4C0d-2>YS2>unclassified family>unclassified genus	0.005	0.004	0.004	0.003	0.004	0.003	0.003	0.004	0.002	0.002	0.007
Fibrobacteres>Fibrobacteria>Fibrobacterales>Fibrobacteraceae>Fibrobacter	0.004	0.003	0.004	0.003	0.005	0.003	0.003	0.001	0.004	0.002	0.098
Firmicutes>Clostridia>Clostridiales>Lachnospiraceae>Pseudobutyrivibrio	0.004	0.004	0.003	0.005	0.004	0.004	0.004	0.004	0.005	0.004	0.843
Firmicutes>Clostridia>Clostridiales>Eubacteriaceae>Pseudoramibacter_Eubacterium	0.004	0.003	0.006	0.006	0.003	0.007	0.002	0.005	0.006	0.004	0.007
Spirochaetes>MVP-15>PL-11B10>unclassified family>unclassified genus	0.004	0.001	0.003	0.001	0.004	<0.001	0.003	0.002	0.002	0.001	0.028
Firmicutes>Clostridia>Clostridiales>Lachnospiraceae>Blautia	0.004	0.004	0.004	0.004	0.004	0.005	0.005	0.004	0.005	0.008	0.365
Bacteroidetes>Bacteroidia>Bacteroidales>Porphyromonadaceae>Paludibacter	0.004	0.007	0.010	0.007	0.003	0.002	0.007	0.007	0.005	0.002	0.699
Firmicutes>Bacilli>Lactobacillales>Lactobacillaceae>Lactobacillus	0.003	0.003	0.005	0.005	0.003	0.004	0.007	0.008	0.005	0.017	0.276
Firmicutes>Clostridia>Clostridiales>Clostridiaceae>Sarcina	0.003	0.001	0.001	0.001	0.001	0.004	0.001	0.002	0.002	0.002	0.493
Firmicutes>Clostridia>Clostridiales>unclassified family>unclassified genus	0.003	0.004	0.005	0.003	0.003	0.004	0.003	0.003	0.003	0.004	0.313
Bacteroidetes>Bacteroidia>Bacteroidales>Bacteroidaceae>Bacteroides	0.003	0.004	0.005	0.007	0.004	0.003	0.003	0.003	0.006	0.003	0.308
Actinobacteria>Actinobacteria>Actinomycetales>Nocardiaceae>Rhodococcus	0.002	0.002	0.002	0.002	0.002	0.003	0.002	0.001	0.002	0.001	0.409
Euryarchaeota>Methanobacteria>Methanobacteriales>Methanobacteriaceae>Methanobrevibacter	0.002	0	0.001	0.001	0.001	0.001	0.001	0.001	<0.001	0.001	0.032
Firmicutes>Erysipelotrichi>Erysipelotrichales>Erysipelotrichaceae>RFN20	0.002	0.001	0.002	0.001	0.001	0.002	0.002	0.002	0.003	0.002	0.851
WPS-2>unclassified class>unclassified order>unclassified family>unclassified genus	0.002	0.001	0.002	0.001	0.001	0.004	0.002	0.002	0.002	0.001	0.049
Bacteroidetes>Bacteroidia>Bacteroidales>Bacteroidaceae>unclassified genus	0.001	0.002	0.004	0.004	0.003	0.002	0.002	0.002	<0.001	0.001	0.001*
Firmicutes>Clostridia>Clostridiales>Lachnospiraceae>Epulopiscium	0.001	<0.001	<0.001	<0.001	0.001	<0.001	<0.001	<0.001	<0.001	<0.001	0.009
Firmicutes>Clostridia>Clostridiales>Lachnospiraceae>[Ruminococcus]	0.001	0.001	0.006	0.001	0.001	0.001	0.004	0.002	0.001	0.001	0.655
Proteobacteria>Gammaproteobacteria>Enterobacteriales>Enterobacteriaceae>unclassified genus	<0.001	<0.001	<0.001	0.001	<0.001	<0.001	<0.001	<0.001	<0.001	<0.001	0.940
Fusobacteria>Fusobacteriia>Fusobacteriales>Fusobacteriaceae>Fusobacterium	<0.001	<0.001	<0.001	0.001	0	<0.001	<0.001	0	<0.001	<0.001	0.859
Verrucomicrobia>Verruco-5>LD1-PB3>unclassified family>unclassified genus	0	<0.001	0	0	0	0	<0.001	0.002	0	0	<0.001*
Other Genera <1%	0.042	0.042	0.044	0.042	0.043	0.055	0.052	0.042	0.047	0.04	0.046

*Level of significance was P≤0.001 after Bonferroni correction for multiple comparisons

Table S6. Comparison of mean relative abundance at phylum level between seasons.

Taxon	Autumn	Spring	Winter	Summer	P value
Firmicutes	0.607	0.666	0.687	0.599	<0.001*
Bacteroidetes	0.234	0.215	0.183	0.244	0.008
Verrucomicrobia	0.042	0.028	0.037	0.040	<0.001*
Unassigned phyla	0.032	0.025	0.023	0.037	<0.001*
Actinobacteria	0.022	0.014	0.016	0.017	0.004
TM7	0.017	0.016	0.019	0.018	0.906
Spirochaetes	0.016	0.011	0.010	0.018	0.009
Proteobacteria	0.007	0.004	0.005	0.007	<0.001*
Tenericutes	0.006	0.005	0.005	0.005	0.752
Fibrobacteres	0.004	0.004	0.002	0.003	0.415
Cyanobacteria	0.004	0.003	0.003	0.004	0.01
Euryarchaeota	0.002	0.001	0.001	0.002	<0.001*
WPS-2	0.002	0.001	0.002	0.001	0.079
Fusobacteria	<0.001	<0.001	<0.001	<0.001	0.107
Other Phyla <1%	0.007	0.007	0.007	0.007	0.636

*Level of significance was P≤0.003 after Bonferroni correction for multiple comparisons

Autumn – March, April, May; Spring – September, October, November; Winter – June, July, August; Summer – December, January, February

Table S7. Comparison of mean relative abundance at genus level between seasons.

Taxon	Autumn	Spring	Winter	Summer	P value
Firmicutes>Clostridia>Clostridiales>Ruminococcaceae>unclassified genus	0.187	0.233	0.226	0.223	<0.001*
Firmicutes>Clostridia>Clostridiales>Lachnospiraceae>unclassified genus	0.144	0.138	0.139	0.121	0.088
Firmicutes>Clostridia>Clostridiales>unclassified family>unclassified genus	0.117	0.134	0.144	0.109	<0.001*
Bacteroidetes>Bacteroidia>Bacteroidales>unclassified family>unclassified genus	0.105	0.101	0.075	0.114	<0.001*
Unassigned genera	0.032	0.025	0.023	0.037	<0.001*
Verrucomicrobia>Verruco-5>WCHB1-41>RFP12>unclassified genus	0.024	0.022	0.016	0.024	<0.001*
Bacteroidetes>Bacteroidia>Bacteroidales>Prevotellaceae>Prevotella	0.024	0.024	0.02	0.018	0.272
Firmicutes>Clostridia>Clostridiales>Ruminococcaceae>Ruminococcus	0.022	0.023	0.024	0.017	0.018
Bacteroidetes>Bacteroidia>Bacteroidales>[Paraprevotellaceae]>unclassified genus	0.021	0.015	0.024	0.017	0.146
Firmicutes>Clostridia>Clostridiales>[Mogibacteriaceae]>unclassified genus	0.019	0.027	0.021	0.021	<0.001*
Verrucomicrobia>Verrucomicrobiae>Verrucomicrobiales>Verrucomicrobiaceae>Akkermansia	0.018	0.006	0.02	0.014	<0.001*
Bacteroidetes>Bacteroidia>Bacteroidales>[Paraprevotellaceae]>CF231	0.017	0.011	0.005	0.01	<0.001*
Bacteroidetes>Bacteroidia>Bacteroidales>[Paraprevotellaceae]>YRC22	0.017	0.015	0.017	0.012	0.384
Bacteroidetes>Bacteroidia>Bacteroidales>BS11>unclassified genus	0.016	0.021	0.02	0.022	0.16
TM7>TM7-3>CW040>F16>unclassified genus	0.016	0.016	0.019	0.017	0.886
Spirochaetes>Spirochaetes>Spirochaetales>Spirochaetaceae>Treponema	0.014	0.01	0.008	0.011	0.06
Firmicutes>Erysipelotrichi>Erysipelotrichales>Erysipelotrichaceae>p-75-a5	0.011	0.013	0.01	0.01	0.035
Firmicutes>Clostridia>Clostridiales>Lachnospiraceae>Coprococcus	0.011	0.011	0.014	0.011	0.051
Firmicutes>Clostridia>Clostridiales>Clostridiaceae>unclassified genus	0.009	0.009	0.014	0.006	<0.001*
Firmicutes>Clostridia>Clostridiales>Veillonellaceae>Phascolarctobacterium	0.009	0.005	0.007	0.01	0.023
Firmicutes>Clostridia>Clostridiales>Clostridiaceae>Clostridium	0.008	0.01	0.007	0.006	<0.001*
Actinobacteria>Coriobacteriia>Coriobacteriales>Coriobacteriaceae>unclassified genus	0.008	0.008	0.008	0.006	0.01
Firmicutes>Clostridia>Clostridiales>Ruminococcaceae>Oscillospira	0.008	0.007	0.01	0.008	0.177
Firmicutes>Bacilli>Lactobacillales>Lactobacillaceae>Lactobacillus	0.007	0.002	0.012	0.002	<0.001*
Bacteroidetes>Bacteroidia>Bacteroidales>Bacteroidaceae>BF311	0.007	0.008	0.006	0.009	0.006
Bacteroidetes>Bacteroidia>Bacteroidales>RF16>unclassified genus	0.006	0.004	0.001	0.012	<0.001*
Firmicutes>Clostridia>Clostridiales>Lachnospiraceae>unclassified genus	0.006	0.007	0.004	0.005	<0.001*
Bacteroidetes>Bacteroidia>Bacteroidales>[Paraprevotellaceae]>[Prevotella]	0.006	0.004	0.004	0.005	0.097
Bacteroidetes>Bacteroidia>Bacteroidales>Porphyromonadaceae>Paludibacter	0.005	0.003	0.001	0.013	<0.001*
Firmicutes>Clostridia>Clostridiales>Christensenellaceae>unclassified genus	0.005	0.007	0.008	0.006	0.005
Firmicutes>Clostridia>Clostridiales>Lachnospiraceae>Pseudobutyrivibrio	0.005	0.004	0.003	0.004	0.279

Tenericutes>Mollicutes>RF39>unclassified family>unclassified genus	0.005	0.004	0.005	0.004	0.343
Firmicutes>Clostridia>Clostridiales>unclassified family>unclassified genus	0.004	0.003	0.004	0.004	0.002
Cyanobacteria>4C0d-2>YS2>unclassified family>unclassified genus	0.004	0.003	0.003	0.004	0.007
Bacteroidetes>Bacteroidia>Bacteroidales>Bacteroidaceae>Bacteroides	0.004	0.004	0.004	0.004	0.014
Firmicutes>Clostridia>Clostridiales>Eubacteriaceae>Pseudoramibacter_Eubacterium	0.004	0.006	0.004	0.005	0.122
Fibrobacteres>Fibrobacteria>Fibrobacterales>Fibrobacteraceae>Fibrobacter	0.004	0.004	0.002	0.003	0.415
Firmicutes>Clostridia>Clostridiales>Lachnospiraceae>Blautia	0.004	0.004	0.004	0.005	0.831
Firmicutes>Clostridia>Clostridiales>Veillonellaceae>unclassified genus	0.003	0.002	0.005	0.001	<0.001*
Firmicutes>Clostridia>Clostridiales>Clostridiaceae>Sarcina	0.002	0.001	0.004	0	<0.001*
Firmicutes>Erysipelotrichi>Erysipelotrichales>Erysipelotrichaceae>RFN20	0.002	0.002	0.001	0.003	<0.001*
Actinobacteria>Actinobacteria>Actinomycetales>Nocardiaceae>Rhodococcus	0.002	0.001	0.001	0.003	<0.001*
Euryarchaeota>Methanobacteria>Methanobacteriales>Methanobacteriaceae>Methanobrevibacter	0.002	0.001	0.001	0.001	0.001*
Bacteroidetes>Bacteroidia>Bacteroidales>Bacteroidaceae>unclassified genus	0.002	0.003	0.002	0.002	0.061
WPS-2>unclassified class>unclassified order>unclassified family>unclassified genus	0.002	0.001	0.002	0.001	0.079
Spirochaetes>MVP-15>PL-11B10>unclassified family>unclassified genus	0.001	0.001	0.001	0.005	0.002
Firmicutes>Clostridia>Clostridiales>Lachnospiraceae>[Ruminococcus]	0.001	0.001	0.005	0.001	0.917
Firmicutes>Clostridia>Clostridiales>Lachnospiraceae>Epulopiscium	<0.001	<0.001	0.001	<0.001	<0.001*
Proteobacteria>Gammaproteobacteria>Enterobacteriales>Enterobacteriaceae>unclassified genus	<0.001	<0.001	<0.001	<0.001	0.019
Fusobacteria>Fusobacteriia>Fusobacteriales>Fusobacteriaceae>Fusobacterium	<0.001	<0.001	<0.001	<0.001	0.056
Verrucomicrobia>Verruco-5>LD1-PB3>unclassified family>unclassified genus	<0.001	<0.001	<0.001	0.001	0.511
Other Genera <1%	0.052	0.039	0.039	0.051	<0.001*

*Level of significance was P≤0.001 after Bonferroni correction for multiple comparisons

Autumn – March, April, May; Spring – September, October, November; Winter – June, July, August; Summer – December, January, February

Table S8. Comparison of mean relative abundance at phylum level between months.

Taxon	Jan	Feb	Mar	Apr	May	Jun	Jul	Aug	Sep	Oct	Nov	Dec	P value
Firmicutes	0.600	0.534	0.508	0.656	0.664	0.677	0.697	0.688	0.668	0.673	0.656	0.662	<0.001*
Bacteroidetes	0.249	0.298	0.328	0.198	0.169	0.179	0.182	0.189	0.220	0.200	0.223	0.185	<0.001*
Unassigned phyla	0.044	0.041	0.042	0.024	0.029	0.023	0.022	0.022	0.022	0.026	0.026	0.025	<0.001*
Verrucomicrobia	0.038	0.041	0.039	0.039	0.050	0.050	0.027	0.032	0.025	0.032	0.028	0.040	0.003*
Spirochaetes	0.018	0.027	0.027	0.012	0.007	0.010	0.009	0.010	0.014	0.009	0.012	0.008	<0.001*
TM7	0.012	0.009	0.005	0.017	0.028	0.018	0.017	0.021	0.014	0.017	0.018	0.032	<0.001*
Actinobacteria	0.010	0.017	0.014	0.029	0.022	0.015	0.020	0.014	0.011	0.016	0.014	0.023	<0.001*
Proteobacteria	0.008	0.009	0.008	0.006	0.006	0.006	0.005	0.003	0.004	0.004	0.003	0.005	<0.001*
Tenericutes	0.005	0.005	0.006	0.004	0.009	0.006	0.004	0.006	0.005	0.005	0.006	0.006	0.009
Cyanobacteria	0.003	0.005	0.005	0.003	0.003	0.002	0.002	0.005	0.003	0.003	0.003	0.004	0.001*
Fibrobacteres	0.002	0.005	0.009	0.002	<0.001	0.002	0.002	0.003	0.006	0.002	0.003	0.001	<0.001*
Euryarchaeota	0.002	0.002	0.001	0.004	0.001	0.001	0.001	0.001	0.001	0.002	0.001	0.001	0.001*
WPS-2	0.002	0.001	<0.001	<0.001	0.004	0.003	0.002	0.001	0.002	0.001	0.001	0.001	0.008
Fusobacteria	<0.001	<0.001	<0.001	<0.001	<0.001	0.001	<0.001	<0.001	<0.001	<0.001	<0.001	<0.001	0.082
Other Phyla <1%	0.008	0.006	0.007	0.007	0.007	0.006	0.008	0.006	0.006	0.008	0.007	0.007	0.206

*Level of significance was P≤0.003 after Bonferroni correction for multiple comparisons

Table S9. Comparison of mean relative abundance at genus level between months.

Taxon	Jan	Feb	Mar	Apr	May	Jun	Jul	Aug	Sep	Oct	Nov	Dec	P value
Firmicutes>Clostridia>Clostridiales>Ruminococcaceae>unclassified genus	0.248	0.186	0.169	0.166	0.230	0.219	0.232	0.228	0.251	0.237	0.212	0.234	<0.001*
Bacteroidetes>Bacteroidia>Bacteroidales>unclassified family>unclassified genus	0.116	0.135	0.157	0.089	0.066	0.063	0.077	0.086	0.108	0.098	0.099	0.093	<0.001*
Firmicutes>Clostridia>Clostridiales>Lachnospiraceae>unclassified genus	0.114	0.118	0.112	0.182	0.137	0.138	0.139	0.140	0.129	0.134	0.153	0.133	0.006
Firmicutes>Clostridia>Clostridiales>unclassified family>unclassified genus	0.100	0.094	0.089	0.135	0.127	0.138	0.149	0.144	0.131	0.136	0.134	0.134	<0.001*
Unassigned genera	0.044	0.041	0.042	0.024	0.029	0.023	0.022	0.022	0.022	0.026	0.026	0.025	<0.001*
Verrucomicrobia>Verruco-5>WCHB1-41>RFP12>unclassified genus	0.028	0.022	0.028	0.024	0.018	0.015	0.014	0.019	0.019	0.026	0.02	0.023	<0.001*
Firmicutes>Clostridia>Clostridiales>[Mogibacteriaceae]>unclassified genus	0.020	0.015	0.014	0.023	0.020	0.018	0.020	0.026	0.024	0.032	0.024	0.029	<0.001*
Bacteroidetes>Bacteroidia>Bacteroidales>RF16>unclassified genus	0.019	0.016	0.016	0.001	0.001	0.001	<0.001	0.003	0.006	0.004	0.003	0.002	<0.001*
Bacteroidetes>Bacteroidia>Bacteroidales>Porphyromonadaceae>Paludibacter	0.019	0.016	0.013	<0.001	<0.001	0.001	0.001	0.003	0.005	0.002	0.001	0.002	<0.001*
Bacteroidetes>Bacteroidia>Bacteroidales>BS11>unclassified genus	0.019	0.024	0.017	0.009	0.025	0.024	0.022	0.015	0.019	0.026	0.019	0.022	0.014
Bacteroidetes>Bacteroidia>Bacteroidales>Prevotellaceae>Prevotella	0.015	0.027	0.038	0.023	0.009	0.019	0.021	0.021	0.020	0.018	0.034	0.013	<0.001*
Bacteroidetes>Bacteroidia>Bacteroidales>[Paraprevotellaceae]>unclassified genus	0.015	0.020	0.021	0.014	0.028	0.026	0.035	0.011	0.016	0.013	0.015	0.016	0.012
Firmicutes>Clostridia>Clostridiales>Ruminococcaceae>Ruminococcus	0.014	0.024	0.024	0.018	0.026	0.028	0.025	0.020	0.023	0.017	0.027	0.014	0.001*
Firmicutes>Clostridia>Clostridiales>Veillonellaceae>Phascolarctobacterium	0.013	0.012	0.015	0.010	0.003	0.005	0.007	0.008	0.007	0.003	0.006	0.005	<0.001*
TM7>TM7-3>CW040>F16>unclassified genus	0.012	0.009	0.005	0.017	0.028	0.018	0.017	0.021	0.014	0.017	0.017	0.031	<0.001*
Bacteroidetes>Bacteroidia>Bacteroidales>[Paraprevotellaceae]>YRC22	0.012	0.016	0.015	0.017	0.019	0.019	0.015	0.016	0.015	0.012	0.017	0.008	0.06
Bacteroidetes>Bacteroidia>Bacteroidales>Bacteroidaceae>BF311	0.011	0.009	0.010	0.003	0.007	0.003	0.003	0.011	0.010	0.007	0.008	0.008	<0.001*
Firmicutes>Clostridia>Clostridiales>Ruminococcaceae>Oscillospira	0.011	0.008	0.007	0.008	0.008	0.008	0.016	0.007	0.009	0.007	0.007	0.007	0.002
Bacteroidetes>Bacteroidia>Bacteroidales>[Paraprevotellaceae]>CF231	0.009	0.011	0.015	0.029	0.004	0.005	0.002	0.009	0.009	0.010	0.014	0.010	<0.001*
Spirochaetes>Spirochaetes>Spirochaetales>Spirochaetaceae>Treponema	0.009	0.017	0.023	0.012	0.006	0.009	0.007	0.008	0.011	0.007	0.010	0.006	<0.001*
Verrucomicrobia>Verrucomicrobiae>Verrucomicrobiales>Verrucomicrobiaceae>Akkermansia	0.009	0.018	0.010	0.014	0.031	0.035	0.012	0.012	0.005	0.005	0.008	0.014	<0.001*

Firmicutes>Clostridia>Clostridiales>Lachnospiraceae>Coprococcus	0.009	0.012	0.008	0.013	0.011	0.014	0.013	0.016	0.013	0.009	0.012	0.013	0.004
Firmicutes>Erysipelotrichi>Erysipelotrichales>Erysipelotrichaceae>p-75-a5	0.008	0.005	0.007	0.013	0.013	0.008	0.010	0.012	0.010	0.019	0.011	0.018	<0.001*
Spirochaetes>MVP-15>PL-11B10>unclassified family>unclassified genus	0.006	0.009	0.003	<0.001	0	0.001	0.002	0.002	0.001	0.001	0.001	0	<0.001*
Firmicutes>Clostridia>Clostridiales>Clostridiaceae>unclassified genus	0.006	0.005	0.005	0.009	0.013	0.020	0.009	0.013	0.010	0.012	0.006	0.008	<0.001*
Firmicutes>Clostridia>Clostridiales>Christensenellaceae>unclassified genus	0.006	0.005	0.005	0.005	0.006	0.005	0.011	0.007	0.006	0.008	0.006	0.008	<0.001*
Firmicutes>Clostridia>Clostridiales>Lachnospiraceae>unclassified genus	0.005	0.005	0.006	0.008	0.004	0.004	0.003	0.006	0.006	0.007	0.008	0.005	<0.001*
Firmicutes>Clostridia>Clostridiales>Lachnospiraceae>Blautia	0.005	0.005	0.003	0.007	0.003	0.005	0.003	0.006	0.005	0.003	0.005	0.006	<0.001*
Bacteroidetes>Bacteroidia>Bacteroidales>[Paraprevotellaceae]>[Prevotella]	0.005	0.009	0.009	0.005	0.003	0.006	0.003	0.004	0.005	0.002	0.004	0.002	<0.001*
Firmicutes>Erysipelotrichi>Erysipelotrichales>Erysipelotrichaceae>RFN20	0.004	0.003	0.006	0.001	<0.001	0.001	<0.001	0.002	0.002	0.002	0.001	0.002	<0.001*
Actinobacteria>Coriobacteriia>Coriobacteriales>Coriobacteriaceae>unclassified genus	0.004	0.004	0.004	0.011	0.010	0.006	0.010	0.009	0.007	0.010	0.007	0.010	<0.001*
Firmicutes>Clostridia>Clostridiales>Clostridiaceae>Clostridium	0.004	0.005	0.008	0.010	0.007	0.005	0.007	0.008	0.009	0.009	0.011	0.007	<0.001*
Bacteroidetes>Bacteroidia>Bacteroidales>Bacteroidaceae>Bacteroides	0.004	0.006	0.007	0.002	0.002	0.009	0.002	0.002	0.004	0.004	0.003	0.003	<0.001*
Firmicutes>Clostridia>Clostridiales>Eubacteriaceae>Pseudoramibacter_Eubacterium	0.004	0.002	0.003	0.005	0.003	0.002	0.003	0.006	0.004	0.010	0.004	0.009	<0.001*
Firmicutes>Clostridia>Clostridiales>unclassified family>unclassified genus	0.004	0.004	0.003	0.005	0.003	0.003	0.004	0.003	0.003	0.003	0.003	0.004	0.001*
Firmicutes>Clostridia>Clostridiales>Lachnospiraceae>Pseudobutyrivibrio	0.004	0.004	0.004	0.007	0.003	0.004	0.002	0.004	0.004	0.004	0.005	0.003	0.046
Firmicutes>Bacilli>Lactobacillales>Lactobacillaceae>Lactobacillus	0.003	0.001	0	0.003	0.019	0.018	0.008	0.009	0.004	0.001	0.002	0.004	<0.001*
Tenericutes>Mollicutes>RF39>unclassified family>unclassified genus	0.003	0.003	0.002	0.003	0.009	0.006	0.004	0.005	0.004	0.004	0.005	0.005	<0.001*
Cyanobacteria>4C0d-2>YS2>unclassified family>unclassified genus	0.003	0.005	0.005	0.003	0.002	0.002	0.002	0.004	0.003	0.003	0.003	0.003	<0.001*
Fibrobacteres>Fibrobacteria>Fibrobacterales>Fibrobacteraceae>Fibrobacter	0.002	0.005	0.009	0.002	0	0.002	0.002	0.003	0.006	0.002	0.003	0.001	<0.001*
Bacteroidetes>Bacteroidia>Bacteroidales>Bacteroidaceae>unclassified genus	0.002	0.003	0.003	0.002	0.001	0.001	<0.001	0.005	0.002	0.003	0.003	0.002	0.002
WPS-2>unclassified class>unclassified order>unclassified family>unclassified genus	0.002	0.001	<0.001	<0.001	0.004	0.003	0.002	0.001	0.002	0.001	0.001	0.001	0.008
Firmicutes>Clostridia>Clostridiales>Clostridiaceae>Sarcina	0.001	<0.001	<0.001	<0.001	0.006	0.005	0.003	0.003	0.001	0.001	0.001	0.001	<0.001*

Actinobacteria>Actinobacteria>Actinomycetales>Nocardiaceae>Rhodococcus	0.001	0.004	0.003	0.002	0.002	0.002	0.001	0.001	<0.001	0.001	0.001	0.003	<0.001*
Firmicutes>Clostridia>Clostridiales>Veillonellaceae>unclassified genus	0.001	0.002	0.002	0.003	0.004	0.005	0.006	0.003	0.002	0.002	0.002	0.001	<0.001*
Firmicutes>Clostridia>Clostridiales>Lachnospiraceae>[Ruminococcus]	0.001	0.001	0.001	0.001	<0.001	0.007	0.007	0.001	0.001	0.001	0.001	0.001	0.026
Firmicutes>Clostridia>Clostridiales>Lachnospiraceae>Epulopiscium	<0.001	<0.001	<0.001	<0.001	<0.001	0.001	<0.001	0.002	<0.001	<0.001	<0.001	<0.001	<0.001*
Euryarchaeota>Methanobacteria>Methanobacteriales>Methanobacteriaceae>Methanobrevibacter	<0.001	<0.001	0.001	0.003	0.001	0.001	0.001	<0.001	0.001	0.001	<0.001	0.001	<0.001*
Proteobacteria>Gammaproteobacteria>Enterobacteriales>Enterobacteriaceae>unclassified genus	<0.001	<0.001	<0.001	<0.001	<0.001	0.001	<0.001	<0.001	<0.001	<0.001	0	<0.001	0.002
Fusobacteria>Fusobacteriia>Fusobacteriales>Fusobacteriaceae>Fusobacterium	<0.001	<0.001	0	0	<0.001	0.001	<0.001	0	0	0	0	<0.001	0.039
Verrucomicrobia>Verruco-5>LD1-PB3>unclassified family>unclassified genus	<0.001	<0.001	<0.001	0	0	0	<0.001	<0.001	0	<0.001	<0.001	0.001	0.879
Other Genera <1%	0.048	0.056	0.051	0.058	0.047	0.038	0.043	0.033	0.035	0.043	0.038	0.048	<0.001*

*Level of significance was P≤0.001 after Bonferroni correction for multiple comparisons

Figure S1. Principal coordinate analysis of the faecal bacterial community by season and month.

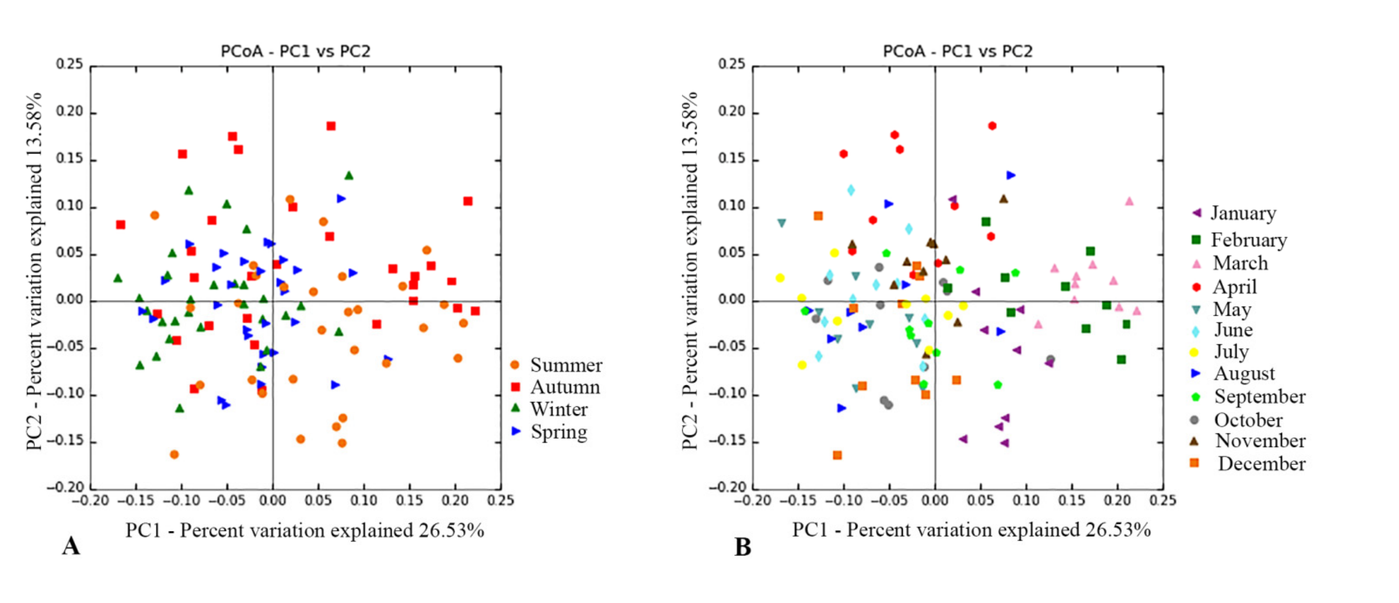

Figure S2. Season-wise hierarchial clustering of the faecal bacterial community of horses included in the study.

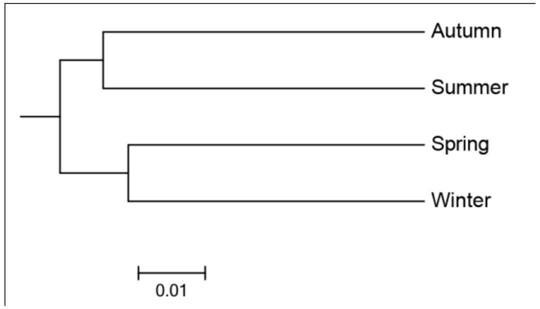

Supplement: Supplementary file 1 [file animals-11-02300-s001.zip › animals-1266668-supplementary.pdf]
